# Effects of Psychological, Environmental and Physical Stressors on the Gut Microbiota

**DOI:** 10.3389/fmicb.2018.02013

**Published:** 2018-09-11

**Authors:** J. Philip Karl, Adrienne M. Hatch, Steven M. Arcidiacono, Sarah C. Pearce, Ida G. Pantoja-Feliciano, Laurel A. Doherty, Jason W. Soares

**Affiliations:** ^1^Military Nutrition Division, U.S. Army Research Institute of Environmental Medicine, Natick, MA, United States; ^2^Soldier Performance Optimization, Natick Soldier Research, Development and Engineering Center, Natick, MA, United States; ^3^Combat Feeding Directorate, Natick Soldier Research, Development and Engineering Center, Natick, MA, United States

**Keywords:** microbiome, stress, nutrition, psychology, physiology, environment, military

## Abstract

Stress, a ubiquitous part of daily human life, has varied biological effects which are increasingly recognized as including modulation of commensal microorganisms residing in the gastrointestinal tract, the gut microbiota. In turn, the gut microbiota influences the host stress response and associated sequelae, thereby implicating the gut microbiota as an important mediator of host health. This narrative review aims to summarize evidence concerning the impact of psychological, environmental, and physical stressors on gut microbiota composition and function. The stressors reviewed include psychological stress, circadian disruption, sleep deprivation, environmental extremes (high altitude, heat, and cold), environmental pathogens, toxicants, pollutants, and noise, physical activity, and diet (nutrient composition and food restriction). Stressors were selected for their direct relevance to military personnel, a population that is commonly exposed to these stressors, often at extremes, and in combination. However, the selected stressors are also common, alone or in combination, in some civilian populations. Evidence from preclinical studies collectively indicates that the reviewed stressors alter the composition, function and metabolic activity of the gut microbiota, but that effects vary across stressors, and can include effects that may be beneficial or detrimental to host health. Translation of these findings to humans is largely lacking at present. This gap precludes concluding with certainty that transient or cumulative exposures to psychological, environmental, and physical stressors have any consistent, meaningful impact on the human gut microbiota. However, provocative preclinical evidence highlights a need for translational research aiming to elucidate the impact of stressors on the human gut microbiota, and how the gut microbiota can be manipulated, for example by using nutrition, to mitigate adverse stress responses.

## Introduction

The human body is host to trillions of microorganisms collectively known as the human microbiota (Huttenhower et al., 2012; Ding and Schloss, 2014). The diversity, composition and function of this community varies across body sites, being shaped predominantly by the unique environmental conditions (e.g., pH, oxygen and substrate availability, moisture level) at different anatomical sites (Costello et al., 2009; Ding and Schloss, 2014). In particular, the GI tract provides an environment uniquely conducive to maintaining a diverse and dense microbial ecosystem, the gut microbiota (Huttenhower et al., 2012), a community which contains a collective genome estimated to be 100 times larger than the human genome (Qin et al., 2010).

Co-evolution with this non-human genome has resulted in a largely mutualistic bi-directional relationship between host and gut microbiota. The host provides a hospitable environment and nutrients, and, in turn, the gut microbiota shapes immune system development and function (Hooper et al., 2012), reinforces the gut barrier (Cani, 2012), metabolizes undigested nutrients and xenobiotics (Holmes et al., 2012), modulates enteric and central nervous system activity (Cryan and Dinan, 2012), and protects against pathogens (Leslie and Young, 2015). However, perturbing the GI environment can initiate a vicious cycle whereby consequent deleterious shifts in the gut microbiota, known as dysbiosis, exacerbate decrements to GI physiology that maintain dysbiosis. Dysbiosis has been associated with transient health decrements including GI permeability and inflammation (Cani et al., 2012; Wells et al., 2017), increased susceptibility to illness and infection (Zanella Terrier et al., 2014; Mackos et al., 2017), and psychological impairments (Cryan and Dinan, 2012; Foster and McVey Neufeld, 2013). Further, dysbiosis has been associated with multiple chronic diseases such as obesity and associated cardiometabolic diseases (Turnbaugh et al., 2009), inflammatory bowel disease (Sartor, 2008), colon cancer (O’Keefe, 2016), autoimmune diseases (Vaahtovuo et al., 2008; Russell et al., 2012), and psychological disorders (Luna and Foster, 2015; Leclercq et al., 2016) among others (Tremaroli and Backhed, 2012). These associations underlie extensive interest in identifying factors causing dysbiosis, and in developing strategies aiming to leverage the gut microbiota’s tremendous genetic potential for health benefit.

It is increasingly recognized that stress modulates gut microbiota community structure and activity, and may be one causal factor in dysbiosis (Mackos et al., 2017). Stress can be defined as a disruption in homeostasis due to environmental, physical, or psychological stimuli (i.e., stressors) that elicits adaptive physiological and behavioral responses to restore homeostasis (i.e., the stress response) (Glaser and Kiecolt-Glaser, 2005). Although stress is a ubiquitous part of daily life for many individuals, military personnel in particular are exposed to unique stressors often at extremes and in combination. These stressors are psychological (e.g., fear, anxiety, cognitive demands), environmental (e.g., climatic extremes, high altitude, noise, pathogens, toxicants, and pollutants), and physical (e.g., strenuous exercise and high energy expenditure, undernutrition, sleep deprivation) (Weeks et al., 2010; Henning et al., 2011), and have been associated with interrelated health decrements including musculoskeletal injury (Jacobs et al., 2014), nutrient insufficiencies (McClung and Gaffney-Stomberg, 2016), endocrine disruption (Nindl et al., 2007; Henning et al., 2011), inflammation (McClung et al., 2013; Pasiakos et al., 2016), immunosuppression (Institute of Medicine, 1999), illness and infection (Connor et al., 2012; Sanchez et al., 2015), and cognitive and psychological impairments (Hoge et al., 2004; Lieberman et al., 2005). Although these decrements are often transient and may build resiliency to combat stress (Dienstbier, 1989), they may also compromise performance, increase attrition, and contribute to the development of chronic health sequelae in some individuals (Hoge et al., 2004; Gaffney-Stomberg and McClung, 2012; Porter et al., 2013).

Growing evidence linking stress to dysbiosis and health decrements suggests that the gut microbiota could be an underappreciated mediator of stress responses and associated sequelae in military personnel. In support, recent studies have begun to link gut microbes and their metabolites with GI permeability, inflammation, GI symptomology, and psychological metrics in military personnel engaged in multiple-stressor training events (Li et al., 2013; Phua et al., 2015; Karl et al., 2017a,b). For example, Karl et al. (2017a) recently reported pronounced changes in gut microbiota composition and gut microbiota-derived metabolites concomitant to increased GI permeability and inflammation during a 4-day military training exercise conducted during the arctic winter. The training environment was characterized by extremely high energy expenditure, insufficient food intake, cold weather, and sleep restriction (Margolis et al., 2016; Karl et al., 2017b), and typified the multiple-stressor environments experienced by military personnel in training and combat. Changes in the relative abundances of >50% of the observed genera within the gut microbiota were reported, and largely demonstrated an enrichment for less dominant taxa at the expense of more dominant taxa during training (Karl et al., 2017a). Further, several associations between the pre-stressed microbiota, and changes in gut microbiota derived metabolites, GI permeability and inflammation were observed (Karl et al., 2017a). In a separate study, Li et al. (2013, 2014) linked subjective ratings of GI distress during combat-training to stress, anxiety, inflammation, and increased intestinal and blood brain barrier permeability. Changes in urinary concentrations of several metabolites potentially derived from the gut microbiota were also observed, and were associated with GI symptomology and GI permeability (Phua et al., 2015). Taken together, these studies provide initial evidence that the gut microbiota may both respond to and influence stress responses during military training and combat.

There is growing recognition that supporting a healthy and resilient gut microbiota may contribute to health and performance optimization in military personnel (Russell and Deuster, 2017; Arcidiacono et al., 2018; Glaven et al., 2018). However, developing recommendations for achieving this aim requires elucidating the impact of military-relevant stressors on the gut microbiota. Toward this aim, we reviewed current literature related to stressor-induced alterations in gut microbiota community structure and metabolic activity. The stressors reviewed include psychological stress, circadian disruption, sleep deprivation, environmental extremes (high altitude, heat, and cold), environmental pathogens, toxicants and pollutants, noise, strenuous physical exertion, and diet. While stressors were selected for their direct relevance to environmental physiology and military personnel (Henning et al., 2011), the selected stressors are not uncommon, alone or in combination, in some civilian populations such as athletes (Clark and Mach, 2016) and first responders (Alexander and Klein, 2009).

## The Gut Microbiota

Resident gut microbes include bacteria, archaea, viruses, and yeast and other fungi whose population densities progressively increase from 10^3^ to 10^4^ cells/mL content in the acidic environment of the stomach to ∼10^11^ cells/mL content in the colon (Sender et al., 2016). Current knowledge of gut microbiota composition and function is predominated by studies targeting bacteria, hence this review centers on the bacterial residents of the gut microbiota. Two phyla, Bacteroidetes and Firmicutes, comprise ∼90% of the average human adult gut microbiota (Huttenhower et al., 2012). High diversity is more apparent at lower taxonomic levels with > 500 different genera and > 1,000 different species having been reported across human populations (Huttenhower et al., 2012; Falony et al., 2016). At the genus level, a “core” human gut microbiota, defined as genera shared by ≥95% individuals, has been recently reported to include only 14 genera (Falony et al., 2016). However, a healthy adult commonly hosts > 100 different bacterial species in their GI tract, with genus and species compositions demonstrating substantial interindividual variation (Qin et al., 2010; Huttenhower et al., 2012). Within this diversity is a tremendous genetic potential that is less variable than composition, indicating substantial functional redundancy within the gut microbiota (Qin et al., 2010; Huttenhower et al., 2012), and that includes myriad functions not found in the human genome (Qin et al., 2010; Huttenhower et al., 2012; Nicholson et al., 2012).

Although there is no consensus on what constitutes a healthy or dysbiotic gut microbiota (Lloyd-Price et al., 2016), there is some level of agreement regarding characteristics deemed either generally favorable or detrimental (**Table 1**). With few exceptions, a more diverse gut microbiota, both in composition and genetic content, is considered a healthier microbiota (Blaser and Falkow, 2009). One reason may be because low-diversity microbiota lack core or “keystone” microbes or microbial genes required to maintain a healthy ecosystem (Petersen and Round, 2014). Linked with diversity as a healthy attribute is the ability of the gut microbiota to resist perturbation or to recover a healthy state following perturbation (Lloyd-Price et al., 2016).

**Table 1:** Putative health-promoting and health-compromising characteristics and functions of the human gut microbiota.

Characteristic	Effect
**Health-promoting**	
High species/genetic diversity	Associated with better health and resilience to perturbation
*Bifidobacterium* (phyla: Actinobacteria), *Lactobacillus* (phyla: Firmicutes)	Genera commonly used in probiotics; linked to multiple favorable health effects including increased resistance to infection and diarrheal disease, immune-enhancement, anti-carcinogenic, vitamin production, and secretion of anti-microbial compounds
*Roseburia, Eubacterium, Clostridium* clusters XIVa and IV (phyla: Firmicutes)	Butyrate producers
*Faecalibacterium prausnitzii* (phyla: Firmicutes)	Anti-inflammatory, butyrate producer
Increased butyrate production	Major energy source of colonocytes, anti-inflammatory, regulates cell growth and differentiation, anti-carcinogenic, improved gut barrier function, reduced colonic pH
Carbohydrate fermentation/increased short-chain fatty acid (butyrate, acetate, propionate) production	Reduced colonic pH, pathogen inhibition, anti-inflammatory, anti-carcinogenic, energy source for peripheral tissues, enhanced mineral absorption
**Health-compromising**	
Low diversity/high pathogen load	Compromised gut barrier integrity, local and systemic inflammation
Proteobacteria (includes family *Enterobacteriaceae*)	Phyla which produces pro-inflammatory lipopolysaccharide
Protein fermentation	Production of potentially carcinogenic/toxic compounds (*N*-nitroso compounds, amines, *p*-cresol, NH_3_, phenols, amines, thiols)
Sulfate/sulfite-reducing bacteria e.g., *Bilophila wadsworthia, Desulfovibrio* (phyla Proteobacteria)	Production of toxic H_2_S
Mucin degradation > synthesis	Compromises gut barrier integrity, facilitates bacterial translocation to epithelium, provides sulfates for H_2_S


A healthy gut microbiota might also be considered a community in which beneficial microbes predominate, while dysbiosis may be characterized by a dominance of one or a few harmful microbes (Roberfroid et al., 2010). Although the health effects of most gut commensals are varied or unclear, there are several taxa generally considered beneficial and several generally considered harmful.

The classic examples of beneficial microbes are the genera, *Lactobacillus* and *Bifidobacterium.* These genera include strains that are commonly used as probiotics, defined as “live microorganisms that, when administered in adequate amounts, confer a health benefit on the host” (Hill et al., 2014). These genera are also the only two historically recognized as beneficial microbes in the prebiotic concept which identifies selective stimulation of *Lactobacillus* and *Bifidobacterium* growth as a health benefit (Roberfroid et al., 2010; Gibson et al., 2017). Strains within these genera enhance immune function, secrete compounds that assist digestion, deter pathogen colonization, and favorably modulate GI physiology (Hill et al., 2014). Notably, recent consensus is that species within the genera *Eubacterium*, *Roseburia*, and *Faecalibacterium* may also be considered beneficial microbes (Roberfroid et al., 2010; Gibson et al., 2017). These taxa produce the SCFA butyrate which has a variety of intraintestinal and extraintestinal health effects to include enhancing gut barrier integrity, and reducing inflammation and oxidative stress (Canani et al., 2011).

At the other end of the spectrum are harmful microbes. Although many commensals would be harmful if they were to enter systemic circulation, and a dominance of any one taxa may be undesirable, Enterobacteriaceae, a family including the gut commensals *Escherichia, Shigella, Proteus*, and *Klebsiella*, have frequently been implicated in the development of inflammation and associated diseases (Huttenhower et al., 2014). Underlying mechanisms include the production of lipopolysaccharide (LPS, also known as endotoxin), a compound attached to the outer cell membrane of gram-negative bacteria which activates the immune system and elicits a strong pro-inflammatory response (Hurley, 1995).

To what extent other taxa are generally beneficial or harmful is less clear. In one comprehensive expert review (Roberfroid et al., 2010), the genera *Staphylococci* and *Veillonella* were characterized as potentially harmful, while others including *Enterococci, Streptococci, Bacteroides, Prevotella, Collinsella*, and *Clostridium*, some of which are abundant gut commensals, were identified as genera containing both potentially beneficial and harmful species. One implication of this uncertainty is that distinguishing beneficial and harmful taxa often requires species-level resolution. Unfortunately, this level of resolution is generally not achieved with high confidence by the high-throughput 16S rRNA gene sequencing approaches currently popular for community-wide analyses of the gut microbiota (Jovel et al., 2016).

Ultimately, the identification of individual taxa as potentially beneficial or harmful is also based on a microbe’s metabolic activity. As such, a healthy gut microbiota might be considered one in which the synthesis of potentially beneficial compounds exceeds that of potentially harmful compounds. Although gut microbes metabolize host-derived compounds (e.g., mucins, sloughed cells), the primary sources of metabolic substrates for the gut microbiota are undigested nutrients from the diet. The metabolites produced by gut microbiota metabolism of these nutrients and their potential health effects have been extensively reviewed (Macfarlane and Macfarlane, 2012; Verbeke et al., 2015), and are only summarized briefly herein.

Undigested carbohydrates are the preferred substrate of many gut microbes, and are fermented by cross-feeding consortia into the SCFA acetate, propionate, and butyrate (Flint, 2012). Beneficial health effects of SCFA include reducing colonic pH and inflammation, stimulating epithelial cell growth, enhancing immunity, deterring carcinogenesis and pathogen colonization, and increasing mineral absorption (Macfarlane and Macfarlane, 2012). Butyrate, in particular, is widely regarded as health promoting. Butyrate is the preferred fuel of colonocytes, has anti-inflammatory, anti-oxidative and anti-neoplastic effects, and improves gut barrier function (Hamer et al., 2008; Macfarlane and Macfarlane, 2012). Recent evidence suggests butyrate may also protect intestinal stem cells from genotoxic compounds in the gut lumen following mucosal damage by reducing stem cell expansion (Kaiko et al., 2016). In contrast, the carbohydrate fermentation intermediates D-lactate and succinate have been associated with dysbiosis, increased GI permeability, and inflammation (Verbeke et al., 2015).

Proteins and amino acids are catabolized by gut microbes into a variety of products including SCFA, BCFA, *p*-cresol, phenolic compounds, hydrogen sulfide, and ammonia (Macfarlane and Macfarlane, 2012; Verbeke et al., 2015). Several of these compounds have demonstrated toxicity and been shown to increase paracellular permeability in *in vitro* cell models although evidence for similar effects at physiologic concentrations *in vivo* is lacking (Verbeke et al., 2015). Other metabolites of amino acid fermentation, such as indolic compounds, may favorably impact the gut barrier (Bansal et al., 2010; Shimada et al., 2013). Gut microbes are also capable of synthesizing neuroactive compounds such as serotonin, dopamine, histamine, and gamma-aminobutyric acid from amino acid precursors (Lyte, 2014). These compounds are thought to impact cognition and behavior via the gut-brain axis (Cryan and Dinan, 2012).

Polyphenols are ubiquitous compounds found in plant foods which have poor bioavailability in the small intestine (Scalbert and Williamson, 2000), but are transformed into a variety of bioavailable compounds by gut microbes (Espin et al., 2017). Some polyphenol metabolites may have prebiotic, anti-inflammatory, anti-oxidative, anti-carcinogenic, and anti-microbial properties (Tuohy et al., 2012), although the function of many remain undetermined.

Lastly, although not a dietary nutrient, bile acids are secreted in response to ingestion of fat. Gut microbes modify bile acids, forming secondary bile acids that act as signaling molecules in multiple metabolic pathways, and which may be health-promoting or health-degrading (Devkota and Chang, 2015; Tran et al., 2015; Wahlstrom et al., 2016).

Taken together, a greater proportion of carbohydrate and plant polyphenol metabolites and some secondary bile acids relative to metabolites of protein fermentation and other secondary or un-modified bile acids may be health-promoting. However, the inability to directly measure production of these compounds in the colon has precluded definitive conclusions regarding health effects, and current consensus is that there is insufficient evidence to consider these compounds either individually or in combination as biomarkers of a healthy or dysbiotic microbiota (Verbeke et al., 2015).

Related to, but separate, from distinguishing healthy and dysbiotic microbiomes is the search for biomarkers within the gut microbiota that may predict response to an intervention, or disease risk. For example, *Prevotella* (reproducibly associated with agrarian, high fiber diets) and *Bacteroides* (reproducibly associated with high-fat, high-protein Western-style diets) have been proposed as possible biomarkers of diet and lifestyle (Gorvitovskaia et al., 2016) that could help predict individual responses to dietary intervention (Kovatcheva-Datchary et al., 2015). *Bacteroides, Escherichia, Acinetobacter, Fusobacterium* and low fecal butyrate concentration have been proposed as potential biomarkers of colorectal cancer risk (Kostic et al., 2012; Ou et al., 2013; O’Keefe, 2016), while depletion of *Faecalibacterium prausnitzii*, impaired butyrate metabolism and an enrichment of Enterobacteriaceae have been identified as potential biomarkers of inflammatory bowel disease (Jansson et al., 2009; Huttenhower et al., 2014). Additional examples include tri-methylamine *N*-oxide, a metabolite derived from bacterial metabolism of dietary choline that has been linked to cardiovascular disease (Wang et al., 2014), and *Eggerthella lenta*, which plays an integral role in mediating effectiveness of the cardiac drug Digoxin (Haiser et al., 2013). Collectively, these examples, and others (Gilbert et al., 2016; Zmora et al., 2016), highlight both the considerable promise for using gut microbiota biomarkers to improve disease risk prediction and inform personalized medicine, and the ultimate value of understanding how the gut microbiota and various stressors interact to impact host physiology.

## Stressors and the Gut Microbiota

### Mechanisms

Although stressors can be varied in nature, the biological stress response is coordinated primarily by the HPA axis and SNS. Stressor-induced activation of the HPA axis and SNS stimulates the release of glucocorticoids, catecholamines, and other hormones (Ulrich-Lai and Herman, 2009) which have varied effects throughout the body including modulation of the immune system and of GI function (Glaser and Kiecolt-Glaser, 2005; Galley and Bailey, 2014). The stress response is largely adaptive and acts to quickly restore homeostasis, but varies as a function of the source, magnitude and duration of stress. Severe or chronic stress can exceed the adaptive capacity of an organism causing reduced physical and cognitive performance, illness, and maladaptive responses leading to disease (Segerstrom and Miller, 2004). A growing body of evidence suggests that host responses to stress may be mediated in part by affecting the gut microbiota.

Several pathways by which stress mediates gut microbiota community structure and activity have been elucidated (Cryan and Dinan, 2012). Specifically, catecholamines and other neuroendocrine hormones directly modulate microbial growth (Lyte and Ernst, 1992), and are secreted by intestinal cells in the GI tract in response to stress (Lyte, 2014). In addition, stress-induced changes in signaling via the vagus nerve and enteric nervous system alter GI motility and reduce digestive activity, likely impacting the gut microbiota by modulating physical forces within the GI tract and by altering substrate availability (Galley and Bailey, 2014). Blood is also redirected away from the GI tract during the stress response, especially in response to vigorous exercise and heat stress, which can initiate a cycle of hypoperfusion, ischemia and reperfusion that alters oxygenation of the GI mucosa, and can create oxidative stress and inflammation (van Wijck et al., 2012). These effects ultimately degrade the physical gut barrier thereby increasing paracellular permeability within the intestinal epithelium (van Wijck et al., 2012). Coinciding changes in oxygenation and metabolic activity within GI microenvironments can impact the gut microbiota (Albenberg et al., 2014). Additionally, it is well established that stress alters immune function (Glaser and Kiecolt-Glaser, 2005). The largest collection of lymphoid tissue in the body, the gut-associated lymphoid tissue, provides a dynamic immunological barrier throughout the GI tract. Changes in the activity of immune cells, epithelial cells, and in the secretion of antimicrobial peptides and other secretory factors within this immunological barrier can directly alter gut microbiota composition and function (Hooper et al., 2012). Finally, environmental factors such as diet, drugs (e.g., antibiotics), pathogens, and environmental toxicants and pollutants may stress the gut microbiota both directly and indirectly via altering inflammation, oxidative stress, immune function, and the GI environment. Diet composition in particular is a major factor influencing the gut microbiota, due to nutrient intake directly affecting the types and nutrients available to gut microbes, and to the myriad effects of different nutrients on host physiology (Ha et al., 2014; Salonen and de Vos, 2014; Louis et al., 2016; Sonnenburg and Backhed, 2016; Yao et al., 2016; Espin et al., 2017; Read and Holmes, 2017).

The effects of individual military-relevant psychological, environmental, and physical stressors on the gut microbiota are reviewed below. For each stressor we briefly describe underpinning mechanisms, and then focus on evidence regarding stressor-induced changes in gut microbiota composition, function and metabolic activity. Because of a relative lack of relevant human studies, evidence from both animal models and experimental human studies is discussed.

### Psychological Stress

Psychological stress has now been associated with multiple GI disorders (Mawdsley and Rampton, 2005; Konturek et al., 2011). Although the underlying causal mechanisms have not been fully elucidated, the association has been attributed to stress-induced alterations in neurohumoral communication between the gut and the brain (i.e., the gut-brain axis) to include altered signaling along the vagus nerve and enteric nervous system, and HPA axis activation resulting in immunomodulation, inflammation, intestinal damage, and increased GI permeability (reviewed in Segerstrom and Miller, 2004; Gareau et al., 2008; Konturek et al., 2011). All of these factors have the potential to influence the gut microbiota.

In support, a growing evidence base links psychological stressors to changes in the murine gut microbiota. Commonly used methods of inducing psychological stress in adult rodents include social defeat/disruption, restraint, and water-avoidance. These models generally induce anxiety-like behaviors, activate the HPA-axis and SNS, induce inflammation, alter GI function and permeability, and modulate immune activity (Gareau et al., 2008) with the magnitude, and, in some cases, the direction of the effect varying with the type and duration of stress used. Given the military perspective of this paper, the social defeat model is of substantial interest. The anxiety-like behavior, social avoidance, and pro-inflammatory state the social defeat model induces is thought to mimic aspects of post-traumatic stress disorder (Hammamieh et al., 2012; Gautam et al., 2015). Several (Galley et al., 2014a,b, 2017a; Golubeva et al., 2015; Gautam et al., 2018), but not all (Bailey et al., 2010, 2011; Aoki-Yoshida et al., 2016; Bharwani et al., 2016; Galley et al., 2017b), studies using these models have reported lower absolute and/or relative abundance of *Lactobacillus* in the murine gut microbiota following stress exposure. This effect is of particular interest because strains within this genus have been shown to enhance immune function, deter pathogen colonization, and favorably modulate GI physiology (Hill et al., 2014). In one study, a single 2-h social disruption was sufficient to change the mucosa-associated microbial community in mice, reducing *Lactobacillus*, and *L. reuteri* in particular (Galley et al., 2014a). Repeated 2-h exposures over 6 days resulted in a greater reduction in *Lactobacillus* (Galley et al., 2014a). Interestingly, reduced *Lactobacillus* abundance has also been documented in infant monkeys exposed to other forms of psychological stress (Bailey and Coe, 1999; Bailey et al., 2004), and recent evidence suggests that translocation of *Lactobacillus* from the intestinal lumen to the spleen may have the beneficial and adaptive effect of priming stress-induced immune activity (Lafuse et al., 2017). Reduced gut microbiota diversity following exposure to social disruption/defeat, restraint stress, and water avoidance stress has also been reported in several (Bailey et al., 2010, 2011; Galley et al., 2014b; Xu D. et al., 2014; Bharwani et al., 2016), but not all (Galley et al., 2014a,b, 2017a,b; Gautam et al., 2018) studies. Reported effects of these stressors on other taxa are less consistent.

An additional military-relevant rodent stress model is that of chronic unpredictable mild stress. This model involves subjecting rodents to multiple psychological, environmental, and physical stressors over several weeks, and has been shown to induce depressive-, anxiety- and despair-like behaviors (Mineur et al., 2006; Bridgewater et al., 2017; Marin et al., 2017). Two recent studies using this model reported reduced *Lactobacillus* abundance in mice exposed to unpredictable mild stress for 3–5 weeks (Bridgewater et al., 2017; Marin et al., 2017). This effect was observed across multiple strains of mice (Marin et al., 2017) and was independent of sex (Bridgewater et al., 2017). In one of those studies, Marin et al. (2017) further demonstrated that restoring *Lactobacillus* via administration of *L. reuteri* ameliorated stress-induced despair behavior. Additional experiments demonstrated that this beneficial effect may be attributable in part to the production of hydrogen peroxide by *Lactobacillus* which can inhibit the conversion of tryptophan to kynurenine (Marin et al., 2017), a compound thought to alter neurotransmitter synthesis and neuroinflammation (Schwarcz et al., 2012). Collectively, these findings suggest a potential causal role for *Lactobacillus* in mitigating stress-induced psychological impairments in mice.

Additional preclinical studies have likewise begun to link psychological stress-induced changes in the gut microbiota to functional consequences in the host. Using an *in silico* approach to predict changes in the genome of the gut microbiota, Bharwani et al. (2016) reported that chronic social defeat stress induced behavioral deficits, immune activation, and increased inflammation while also reducing compositional and genetic diversity within the fecal microbiota. This included reduced relative abundance of genes within pathways involved in the biosynthesis and metabolism of fatty acids (e.g., SCFA) and the amino acid neurotransmitter-precursors tryptophan and tyrosine (Bharwani et al., 2016), implying a reduction in the gut microbiota’s capacity to produce beneficial SCFA and neurotransmitters. Using a restraint stress model, Galley et al. (2017b) demonstrated that stress altered murine gut microbiota composition by depleting *Bifidobacterium*, a beneficial genus. Germ free mice that were colonized with the microbiota of the stress-exposed, *Bifidobacterium*-depleted mice exhibited a heightened pro-inflammatory response and worse colonic pathology when infected with the pathogen *Citrobacter rodentium* relative to infected mice colonized with the microbiota of non-stressed donors (Galley et al., 2017b). Finally, Gao et al. (2018) reported that chronic restraint stress increased the severity of experimentally induced colitis in mice and altered gut microbiota composition by increasing pro-inflammatory bacteria and reducing abundance of the butyrate-producing family Lachnospiraceae. Both co-housing stressed and unstressed mice, and antibiotic treatment mitigated these effects (Gao et al., 2018). Importantly, these latter two studies suggest that stress-induced alterations in the gut microbiota may increase susceptibility to the deleterious effects of subsequent stressors.

The effects of psychological stress on the human gut microbiota are largely unexplored (**Table 2**). One observational study reported increased stress and reduced abundance of fecal lactic acid bacteria (which include *Lactobacillus*) in undergraduates during a week of exams (Knowles et al., 2008). However, while a stress-induced reduction in *Lactobacillus* is consistent with several animal studies, the observational design of that study precluded attributing changes in lactic acid bacteria abundance to the stress of taking exams. No changes in gut microbiota composition were observed in a similar study of students taking medical exams (Kato-Kataoka et al., 2016).

**Table 2:** Longitudinal studies examining effects of military-relevant stressors on human gut microbiota composition and metabolites.

Reference	Design	Microbiota method	Results- Microbiota	Results- Microbiota metabolites
**Military training**				
Karl et al., 2017a	*n* = 26M; Norwegian soldiers before and after 4-day arctic military training	16S rRNA gene sequencing	Changes in relative abundance of 58% of genera (e.g., ↑*Peptostreptococcus*. *Christensenella, Staphylococcus, Bulleidia, Peptoniphilus, Acidaminococcus, Fusobacterium*; ↓*Faecalibacterium, Roseburia, Bacteroides, Collinsella*) ↑Diversity due to increased abundance of less dominant taxa	Fecal metabolome: Changes in microbiota linked to changes in 69 metabolites affected by training; e.g., ↓secondary bile acids, amino acid fermentation metabolites; ↑*p*-cresol
				Plasma metabolome:Changes in microbiota linked to changes in 30 metabolites affected by training; e.g., ↑amino acid fermentation metabolites; ↓benzoate metabolites; ↑↓secondary bile acids
**Psychological stress**				
Knowles et al., 2008	*n* = 23 (7M); measured before and during exams	Targeted; culture	↓Lactic acid bacteria post-exam	NA
Kato-Kataoka et al., 2016	*n* = 46 (25M); measured before and during medical exams, randomized to placebo or probiotic	16S rRNA gene sequencing	No reported effects of stress	NA
**Circadian disruption/Sleep restriction**				
Benedict et al., 2016	*n* = 9M; 2 nights 4.25 h SO vs. 2 nights 8.5 h SO	16S rRNA gene sequencing	↓Tenericutes	*↔*Fecal SCFA, BCFA
			↑Firmicutes:Bacteroidetes ratio, Coriobacteriaceae, Erysipelotrichaceae	
Zhang et al., 2017	*n* = 11 (6M); 5 nights of 4 h SO followed by 2–5 nights of 12 h SO	16S rRNA gene sequencing	No effects	NA
**High Altitude**				
Kleessen et al., 2005	*n* = 7 (5M); 47 days expedition to 6677 m	Targeted; FISH	↑Gammaproteobacteria, Enterobacteriaceae ↓*Bifidobacterium, Atopobium/Coriobacterium/Eggerthella lenta* *↔*Total bacteria, *C. coccoides/E. rectale, Lactobacillus/Enterococcus, C. hitolyticum, Bacteroides, F. prausnitzii*	NA
Adak et al., 2013	*n* = 15M; 15 days at 3505 m	Targeted; culture	↑Total anaerobes, *Escherichia coli, Bacteroides, C. perfringens, Bifidobacterium, Lactobacillus, Peptostreptococcus*, proteinase producers, amylase producers, tannase producers	↓Fecal α-amylase activity↑Fecal proteinase, β-gluronidase, alakaline phosphatase activity
			↓Total aerobes, phosphatase producers	
Sket et al., 2017a,b, 2018	*n* = 11M; 21 days bed rest at normobaric hypoxia (∼4000 m) vs. 21 days bed rest at normoxia	Targeted; qPCR 16S rRNA gene sequencing; SM	↑*Bacteroides* spp.; *↔Roseburia/E. rectale, F. prausnitzii*, butyrate producing communities	*↔*Fecal pH, SCFA, BCFA, fecal metabolome
***Heat and cold:*** No human studies				
***Toxicants/pollutants:*** No human studies				
***Noise:*** No human studies				
***Travelers’ diarrhea***				
Kampmann et al., 2016	*n* = 13 (3M) adults testing positive for *Campylobacter jejuni* or *Salmonella enterica* after traveling to high risk region for 1–3 weeks; no Abx use	16S rRNA gene sequencing	No changes in relative abundance of any taxa over time	NA
Pop et al., 2016	*n* = 12 (7M) adults challenged with ETEC + ciprofloxacin who developed diarrhea (*n* = 5) or did not (*n* = 7)	16S rRNA gene sequencing	Diarrhea vs. no-diarrhea post infection: Transient ↓diversity; ↑*Escherichia* (largely attributed to the challenge strain); within diarrhea group post-infection community similar to pre-treatment community by day 28	NA
**Physical activity**				
Allen et al., 2018b	*n* = 32 (12M; 14 obese); 3 days/week moderate exercise for 6 weeks then sedentary washout for 6 weeks	16S rRNA gene sequencing	Lean-exercise: ↓*Bacteroides*; ↑*Faecalibacterium, Lachnospira*, SCFA-regulating genes	Lean-exercise: ↑Fecal acetate, propionate, butyrate
			Lean-washout: ↑*Collinsella, Dorea;* ↓*Faecalibacterium, Lachnospira*, SCFA-regulating genes	Lean-washout: ↓Fecal propionate, butyrate
			Obese-exercise: ↑*Bacteroides, Collinsella*; ↓*Faecalibacterium; ↔*relative abundance of SCFA-regulating genes	Obese-exercise: ↔Fecal acetate, butyrate, propionate
			Obese-washout: ↓*Collinsella, Dorea*, SCFA-regulating genes*;* ↑*Faecalibacterium, Lachnospira*	Obese-washout: ↔Fecal acetate, butyrate, propionate


*Abx, antibiotics; BCFA, branched-chain fatty acids; ETEC, enterotoxigenic *Escherichia coli*; FISH, fluorescence in-situ hybridization; M, men; SCFA, short-chain fatty acids; SM, shotgun metagenomics; SO, sleep opportunity; ↑, increase/higher; ↓, decrease/lower; ↔, no change/no difference. Genus abbreviations: *C., Clostridium; E.*, *Eubacterium; F; Faecalibacterium.**

In summary, current evidence indicates that psychological stress induces myriad physiologic effects that could influence the gut microbiota. Animal studies report stress-induced changes in gut microbiota composition that while varied, have frequently included reduced *Lactobacillus* abundance, and less frequently reduced diversity. The functional implications for the host are unclear, but may include psychological impairments mediated, in part, by altered tryptophan metabolism and increased susceptibility to subsequent stressors. Additional effects could include altered cognition and behavior as it is increasingly evident that the relationship between brain, gut, and gut microbiota, known as the gut microbiota-gut-brain axis, is bi-directional (reviewed in Cryan and Dinan, 2012; Kelly et al., 2015; Forsythe et al., 2016). As discussed above, stress-induced activation of the SNS and HPA-axis have varied effects on the GI tract and, likely, the gut microbiota. The gut microbiota, in turn, modulates gut barrier integrity, inflammation and immune function, and synthesizes, or stimulates the endogenous secretion of myriad compounds including hormones, neurotransmitters (e.g., serotonin, dopamine, histamine, and gamma-aminobutyric acid), and SCFA. These actions are thought to alter central nervous system activity via a combination of signaling through the enteric nervous system, and spinal and vagal nerves, and, possibly, through direct effects in the brain after passage into circulation and through the blood brain barrier (Kelly et al., 2015; Forsythe et al., 2016). However, the extent to which the gut microbiota-gut-brain axis, and modulation of the gut microbiota through this axis influences host cognition and behavior remains to be determined. This is particularly true for the human host, in whom relationships between psychological stress, the gut microbiota, and subsequent effects on cognition and behavior are underexplored.

### Circadian Disruption and Sleep Restriction

#### Circadian Disruption

Circadian rhythms are the endogenous ∼24 h rhythmic patterns displayed by most organisms, and are central mediators of physiology and behavior (Voigt et al., 2016a; Thaiss et al., 2017). Circadian rhythms are primarily controlled by the core molecular clock, which modulates the activity of transcription factors that regulate expression of clock-controlled genes found within most host cells (Voigt et al., 2016a). Disruption of this loop can be caused by factors that disrupt light-dark cycles such as shift work, rotating work and social schedules. Within the GI tract, variable feeding schedules and diet composition have been shown to disrupt circadian rhythms as well (Asher and Sassone-Corsi, 2015). The health effects of circadian disruption are increasingly recognized, and include both short and long term health decrements such as increased GI permeability (Summa et al., 2013; Voigt et al., 2016b), altered immune responses (Curtis et al., 2014), increased susceptibility to inflammation and GI epithelium damage (Pagel et al., 2017), and multiple chronic inflammation-associated diseases including irritable bowel syndrome and inflammatory bowel disease (Hoogerwerf, 2009; Voigt et al., 2016a; James et al., 2017). Increasingly, evidence suggests that these effects may be mediated in part by the gut microbiota.

The murine gut microbiota, its genome, and its biogeography show diurnal rhythmicity that appears to be driven largely, but not completely (Leone et al., 2015), by host eating behavior (Thaiss et al., 2014, 2016; Zarrinpar et al., 2014; Liang et al., 2015). For example Zarrinpar et al. (2014) reported diurnal fluctuations in the abundance of putatively beneficial microbes (e.g., *Lactobacillus, Lactococcus, Oscillibacter*) as a function of feeding patterns, while others have reported metabolites such as SCFA exhibit diurnal variability (Leone et al., 2015), and may regulate extra-intestinal clocks (Tahara et al., 2018). Of particular interest is reported diurnal oscillations in the abundance of mucus degrading taxa, their genes, and their adherence to the GI mucus layer that parallel fed/fasting cycles (Thaiss et al., 2014, 2016; Zarrinpar et al., 2014; Liang et al., 2015). The result is that exposure of the GI epithelium to gut microbes follows a rhythmic pattern, at least in mice, and evidence suggests that host-microbe crosstalk appears to oscillate in parallel (Thaiss et al., 2016).

Disrupting host circadian rhythms mostly abolishes rhythmicity in the gut microbiota and its genome, and alters gut microbiota composition and metabolic activity with potentially deleterious health effects (Thaiss et al., 2014, 2016; Liang et al., 2015). In support, genetic knockout models have been used to demonstrate an expansion of potentially pro-inflammatory taxa including Rikenellaceae and Clostridiaceae (Liang et al., 2015), and reduced microbiota diversity (Voigt et al., 2016b) during circadian disruption. Interestingly, circadian disorganization induced by manipulating light/dark cycles had no effect on the gut microbiota in mice fed standard chow diets, but exacerbated community changes induced by high-fat, high-sugar diets by promoting an increased relative abundance of the mucin-degrading genus *Ruminococcus* and decreased relative of abundance of anti-inflammatory *Lactobacillus* which was interpreted as a potential mechanism by which circadian disruption could promote intestinal barrier dysfunction and inflammation (Voigt et al., 2014). Thaiss et al. (2014) demonstrated that both ablating the circadian clock and manipulating light/dark cycles mostly abolished normal rhythmicity in gut microbiota composition and its genome, and reduced gut microbiota diversity. Germ free mice colonized with the disrupted microbiota demonstrated impaired glucose tolerance and excess weight gain (Thaiss et al., 2014). In a subsequent study, the same group demonstrated that circadian rhythmicity in the localization of bacteria within the GI tract and in their metabolic activity modulates host gene expression and metabolite profiles (Thaiss et al., 2016). Abolishing this rhythmicity resulted in altered hepatic and intestinal gene expression, and impaired hepatic drug metabolism in conventional mice, but not germ free or antibiotic treated mice. Hepatic gene expression and metabolism has also been linked to the gut microbiota in related studies that have demonstrated the gut microbiota and its metabolites regulate homeostatic circadian hepatic functions (Murakami et al., 2016). These observations imply a role for the gut microbiota in modulating circadian variation in hepatic metabolism of drugs, and likely other dietary and xenobiotic compounds. These findings may prove to have important implications for the timing of interventions targeting the gut microbiota, and elucidating functional consequences of microbe rhythmicity on host physiology. Recent evidence suggests that one functional consequence may include modulation of host energy storage and body composition (Wang et al., 2017).

To what extent circadian disruption impacts the human gut microbiota is largely unexplored. Variations in human gut microbiota composition and related metabolites (butyrate and propionate) were recently associated with time of day (Kaczmarek et al., 2017), and one small human study reported diurnal oscillations in ∼10% of operational taxonomic units (OTUs) identified in the gut microbiota of two adults (Thaiss et al., 2014). Interestingly, colonization of germ free mice with the jet-lagged microbiota collected from two adults resulted in impaired glucose tolerance and excess body fat gain compared to controls which resolved following recovery from jet lag (Thaiss et al., 2014). Although intriguing, those results warrant cautious interpretation given the small sample size.

In summary, the murine gut microbiota, and possibly the human gut microbiota, exhibit diurnal oscillations that appear to be largely associated with feeding and fasting cycles, and, possibly, diet composition. Disruption of this rhythmicity may have deleterious effects on the gut microbiota resulting in alterations in host–microbe crosstalk that impact host gene expression, and physiology. However, the evidence base is limited to animal models and translation to humans is needed.

#### Sleep Restriction

Sleep restriction has been associated with several physiologic effects that could alter the GI environment and hence impact the gut microbiota. First, inadequate sleep (<7 h/night) is thought to activate a classical stress response as evidenced by increased HPA-axis activity and cortisol release, although this response has not been observed in all studies (reviewed in Reynolds et al., 2017). Second, rodent models of sleep deprivation have demonstrated increased oxidative damage and cell death in the intestine (Everson et al., 2014), in addition to infection of body tissues with pathogenic bacteria found in the intestine (Everson and Toth, 2000). The latter finding suggests that immunosuppression and gut barrier dysfunction may result from sleep deprivation, and facilitate bacterial translocation from the gut lumen into systemic circulation (Everson and Toth, 2000). The same effects could also impact gut microbiota composition and activity.

Few studies have examined the effects of sleep restriction on the gut microbiota, and results of those that have are inconsistent. In rats, intestinal overgrowth of total aerobes, and total facultative anaerobes, including several pro-inflammatory and pathogenic species, was documented following 10 days of near total sleep deprivation (Everson and Toth, 2000). More recently, 4 weeks of sleep fragmentation in mice was associated with changes in gut microbial community structure, increased relative abundances of Firmicutes, Lachnospiraceae and Ruminococcaceae, and decreased relative abundances of Bacteroidetes, Actinobacteria, Lactobacillaceae, and Bifidobacteriaceae (Poroyko et al., 2016). Of note, colonizing germ free mice with the gut microbiota of mice exposed to sleep fragmentation resulted in increased plasma LPS-binding protein concentrations and inflammation (Poroyko et al., 2016) implicating a role for the gut microbiota in the metabolic dysfunction associated with chronic inadequate sleep (reviewed in Knutson et al., 2007; Schmid et al., 2015). In both studies, sleep disrupted animals consumed more food than controls (Everson and Toth, 2000; Poroyko et al., 2016). Increased food consumption may therefore comprise an indirect mechanism by which sleep restriction impacts the gut microbiota, and may explain Poroyko et al. (2016) observations that taxa known to feed on undigested nutrients (Lachnospiraceae and Ruminococcaceae), were increased following sleep fragmentation. In contrast, minimal changes in gut microbiota composition were observed in a separate study of *ad libitum* fed (food intake not reported) sleep restricted (4 h/night for 7 days) rats (Zhang et al., 2017).

Results from human studies are scarce and similarly inconsistent (**Table 2**). In healthy young men fed a controlled diet, 2 days of partial sleep deprivation (4.25 h/night) resulted in an increase in the Firmicutes:Bacteroidetes ratio, a decrease in the relative abundance of Tenericutes, and increased relative abundances of Coriobacteriaceae and Erysipelotrichaceae in fecal samples, but no change in fecal SCFA concentrations (Benedict et al., 2016). The authors noted that similar compositional changes have been associated with metabolic perturbation in animal and human studies. In contrast, Zhang et al. (2017) did not observe any changes in gut microbiota composition in *ad libitum* fed adults given a sleeping opportunity of 4 h/night for 5 days. Thus, although effects of sleep restriction on both the murine and human gut microbiota are plausible, and suggested by some studies, the evidence base is small and has not clearly separated any direct physiologic effects of sleep restriction on the gut microbiota from changes in eating behaviors.

### Environmental Stressors

#### High Altitude

Common sequelae of high altitude (≥2500 m) exposure include GI symptoms such as appetite loss, indigestion, nausea, vomiting, gas, and abdominal pain which are attributable in part to the hypobaric hypoxia of high altitude (Anand et al., 2006). Hypobaric hypoxia is characterized by a decrease in the partial pressure of inspired oxygen in proportion to elevation which ultimately results in a drop in arterial oxygen saturation that subsequently reduces delivery of oxygen to peripheral tissues. This may alter GI motility and induce oxidative stress and inflammation (Dosek et al., 2007). On the other hand, intestinal epithelial cells operate under a steep oxygen gradient under normal physiologic conditions, and may therefore be more resistant to the stress of hypobaric hypoxia relative to other tissues (Colgan and Taylor, 2010).

Few studies have examined the effects of hypobaric hypoxia on gut microbiota composition. In rats, exposure to hypobaric hypoxia has been associated with physical decrements in intestinal morphology (Zhou et al., 2011; Adak et al., 2014; Xu C.L. et al., 2014; Zhang F. et al., 2015), oxidative stress (Zhou et al., 2011; Adak et al., 2014; Xu C.L. et al., 2014), inflammation (Xu C.L. et al., 2014), increased serum endotoxin concentrations and bacterial translocation (Zhou et al., 2011), and changes in gut microbiota composition (Adak et al., 2014; Xu C.L. et al., 2014). However, none of these studies have been able to definitively separate the effects of hypobaric hypoxia from the underfeeding and weight loss characteristic of exposure to hypobaric hypoxia.

Human studies conducted in high altitude environments have been observational and likewise unable to separate effects of hypobaric hypoxia from potentially confounding factors such as dehydration, foodborne pathogens, undernutrition, and increased physical activity (**Table 2**). Nonetheless, increased abundance of pro-inflammatory Enterobacteriaceae in association with increased inflammation, and decreased abundance of *Bifidobacterium* were reported during one mountaineering expedition in the Himalaya mountains (Kleessen et al., 2005). In a study of soldiers sojourning at 3505 m, Adak et al. (2013) reported decreased total aerobe counts, and increases in several beneficial (*Bifidobacterium*, and *Lactobacillus*) and several potentially harmful (*Escherichia coli, Clostridium perfringens*) anaerobes. However, little detail was provided with respect to diet, activity or other environmental conditions. Of some relevance are recent reports that provided comprehensive insight into the independent effects of hypoxia on the gut microbiota by comparing subjects maintained on bed rest under normoxic or normobaric hypoxic (∼4000 m simulated altitude) conditions for 21 days (Sket et al., 2017a,b, 2018). That study reported a hypoxia-induced enrichment of *Bacteroides* relative abundance (Sket et al., 2017b, 2018) and of bacterial genes related to iron metabolism, virulence and mucin degradation (Sket et al., 2018), but little effect on the microbial metabolome (Sket et al., 2017a, 2018). Unfortunately, these findings cannot be extrapolated to high altitude environments as some of the hypoxia-mediated effects were mitigated when subjects were not confined to bed rest, and due to uncertainty regarding whether normobaric hypoxia fully reproduces the physiologic effects of hypobaric hypoxia (Millet et al., 2012).

Collectively, these studies suggest that high altitude expeditions are associated with increases in abundance of pro-inflammatory taxa, while associations with potentially beneficial taxa are inconsistent. However, the evidence base is sparse and limited. Randomized, controlled trials are needed to determine the independent effects of hypobaric hypoxia on the gut microbiota, and the subsequent implications for health and performance.

#### Cold

Acute cold exposure elicits multiple physiologic responses that collectively serve to maintain body temperature within the normal physiologic range. Responses include activation of the SNS, cutaneous vasoconstriction which helps insulate the body’s core, and increased skeletal muscle contractile activity which increases metabolic heat production (Castellani and Young, 2016). In rodents, combining acute cold stress with psychological stress (i.e., restraint) is an established model for rapidly inducing gastric ulcers (Senay and Levine, 1967), and has been shown to increase GI permeability (Saunders et al., 1994; Coskun et al., 1996). To our knowledge, similar responses have not been reported in humans, although vasoconstriction of the mesenteric artery during acute cold exposure has been suggested (Wilson et al., 2007). Thus, SNS activation and alterations in intestinal barrier homeostasis could impact gut microbiota during cold exposure.

Recent evidence suggests that cold exposure induces alterations in the murine gut microbiota which may, in turn, promote physiologic adaptations to cold in the host. In mammals, physiologic adaptations following repeated or chronic cold exposure include a blunted physiologic response to cold, enhanced heat conservation, and/or a more pronounced thermogenic response (Castellani and Young, 2016). An increase in intestinal absorptive capacity following cold exposure has also been reported in animals (Toloza et al., 1991), and is thought to facilitate increased energy uptake to support the elevated metabolic rate supporting thermogenesis. These adaptations may be facilitated in part by the gut microbiota (Chevalier et al., 2015; Zietak et al., 2016). Specifically, Chevalier et al. (2015) reported that the gut microbiota of mice exposed to cold for 11–31 days did not demonstrate changes in community diversity. However, the gut microbiota of those mice did show changes in the relative abundances of multiple taxa, several of which mirrored aspects of microbiotas previously associated with obesity (e.g., increased Firmicutes:Bacteroidetes ratio and decreased *Akkermansia muciniphila* [phyla Verrucomicrobia] abundance). Colonizing germ free mice with the cold-exposed microbiota enhanced energy harvest from the diet by increasing both SCFA production and absorptive capacity of the small intestine, and promoted browning of adipose tissue to support increased thermogenesis (Chevalier et al., 2015). Zietak et al. (2016) reported that the gut microbiota of mice exposed to cold for 1—6 days demonstrated decreased community diversity, a decreased Firmicutes:Bacteroidetes ratio, and reduced Verrucomicrobia relative abundance in addition to changes in the abundance of several other taxa. Transfer of the cold-exposed microbiome to germ free mice altered bile acid metabolism, promoted increased thermogenesis, and protected against diet-induced obesity (Zietak et al., 2016). Despite inconsistencies in the taxonomic effects reported in these two studies which may be attributable to differences in dietary intake and/or the duration of cold exposure, both demonstrated that the gut microbiota contributes to cold adaptation in mice. Intriguingly, these studies suggest that any effects of cold exposure on the human gut microbiome could be beneficial by promoting cold tolerance. However, to our knowledge, the effects of cold exposure on the human gut microbiota, and whether any effects contribute to physiologic adaptations to cold have not been explored.

#### Heat Stress

The mechanisms underlying detrimental effects of heat stress on gut barrier function have been expertly reviewed (Lambert, 2008; Dokladny et al., 2016) and studied in a variety of human and animal models. As little as 4–6 h of exposure can have severe deleterious effects on the intestinal epithelium (Pearce et al., 2014). Further, hyperthermia of the intestinal wall can result in damage to the gut barrier and increase permeability of tight junctions (Zuhl et al., 2014) and potentially cause inflammation as well as sepsis (reviewed in Lambert, 2008).

Several animal studies have documented changes in the gut microbiota due to environmental heat stress. Changes included reduced gut microbiota diversity (Sohail et al., 2015), and reduced *Lactobacillus* and *Bifidobacterium* abundance in chickens (Song et al., 2014; Sohail et al., 2015). Those observations suggest that environmental heat stress may have deleterious effects on the gut microbiota. However, to our knowledge, no studies have examined acute or prolonged heat stress on the human gut microbiota.

#### Enteric Pathogens

Acute infectious diarrhea is considered a major public health issue in both developed and developing nations due to the myriad infectious bacteria, viruses and parasites that can be transmitted through foodborne and other environmental vectors (Riddle et al., 2016). In military personnel, acute diarrhea during deployment or TD historically has been, and remains, one of the most common illnesses and causes of non-battle injury. This is especially true of deployments to developing countries where self-reported incidences of TD average 30% (Porter et al., 2017), and in some reports > 50% (Sanders et al., 2004; Riddle et al., 2008). Common causative agents of TD are bacteria, and include enteroaggregative and enterotoxigenic *E. coli* [ETEC], *Campylobacter jejuni, Shigella* spp., and *Salmonella* spp. (Porter et al., 2017). These pathogens elicit diarrhea through diverse mechanisms including immune dysregulation and physical disruption of the gut barrier which ultimately perturb the GI environment (Navaneethan and Giannella, 2008). Treatment of TD commonly includes administration of one or more antibiotics (Riddle et al., 2017) which target pathogens, but also some commensal gut microbes (Ferrer et al., 2017). Thus, both the pathogen and its eradication impact the commensal gut microbiota. Although TD and other infectious diarrhea usually resolves within a week, a significant number of individuals experience post-infectious GI issues (Porter et al., 2011). For example, 5–10% report post-infectious irritable bowel syndrome (Halvorson et al., 2006; Schwille-Kiuntke et al., 2015). Although the underlying etiologies of post-infectious GI disorders are unclear, persistent dysbiosis within the gut microbiota is one possible factor (Riddle and Connor, 2016).

Few studies have examined changes in the gut microbiota during or following TD. In one cross-sectional study, gut microbiota composition after returning from travel was associated with both the presence of TD during travel and the causative pathogen (Youmans et al., 2015). However, two small longitudinal studies reported no persistent changes in gut microbiota composition following *Campylobacter jejuni, Salmonella enterica*, or ETEC infection (Kampmann et al., 2016; Pop et al., 2016), although the ETEC study did report transient proliferation of the pathogenic *E. coli* strain and decreased community diversity that resolved within 28 days of infection (Pop et al., 2016). Importantly, both studies provided evidence suggesting that gut microbiota composition prior to pathogen exposure may be associated with infection risk. Although not a study of TD *per se*, David et al. (2015) recently used a combination of 16S rRNA gene sequencing and shotgun metagenomics to derive a four-step model explaining gut microbiota community dynamics following *Vibrio cholerae* and ETEC infection and treatment in residents of Bangladesh. The model proposed that initial stages of infection and antibiotic treatment reduce abundance of obligate and facultative anaerobes allowing oxygen and dietary/host substrates (i.e., polysaccharides) to accumulate in the gut. Initial recolonization by facultative anaerobes (e.g. *Escherichia, Enterococcus*) then lowers oxygen tensions allowing for obligate anaerobes (e.g., *Bacteroides*) to repopulate. These processes may be aided by phages targeting the initial post-infection colonizers. Subsequent competition for dietary and host substrate then restores the community to a more normal state, although it could not be determined from that study if the normal state matched the pre-infection community structure (David et al., 2015). Whether similar dynamics and mechanisms characterize recovery from infectious diarrhea caused by other pathogens is unclear. Taken together, these studies highlight the need for more research regarding the impact of TD on the gut microbiota, particularly in those who develop post-infectious GI disorders, and suggest that both individual differences and the causative agents will need to be considered.

An additional consideration is the differential effects of antibiotics commonly used to treat TD on the gut microbiota. Very generally antibiotics induce a stress response within the gut microbiota (Maurice et al., 2013) that ultimately leads to reduced gut microbiota diversity and increased susceptibility to pathogen colonization (reviewed in Modi et al., 2014; Ferrer et al., 2017). However, at the taxonomic level it is well established that separate antibiotics differentially affect the gut microbiota (Ferrer et al., 2017). Antibiotics currently recommended for treating deployment-associated TD include azithromycin, levofloxacin, ciprofloxacin, and rifaximin (Riddle et al., 2017; Tribble, 2017). All are thought to affect various commensal microbes (Ferrer et al., 2017). Ciprofloxacin in particular has widespread effects on the human gut microbiota. Reduced diversity and decreased relative abundances or elimination of multiple beneficial taxa including *Bifidobacterium* and several butyrate-producers has been shown to persist for several weeks to a year following ciprofloxacin use in healthy adults (Dethlefsen and Relman, 2011; Rashid et al., 2015; Zaura et al., 2015). In contrast, effects of levofloxacin appear to be limited to *Escherichia* and *Staphylococcus* (Ferrer et al., 2017), and rifaximin may have few effects on gut microbiota community structure, but promote increases in *Lactobacillus* and *Bifidobacterium* while suppressing Proteobacteria (reviewed in DuPont, 2016; Ponziani et al., 2016). Azithromycin has been shown to transiently reduce gut microbiota diversity in healthy adults, with taxonomic effects largely limited to reductions in the relative abundances of only a few families within the Firmicutes phylum (Abeles et al., 2016), but may have more pronounced, potentially deleterious and long-lasting effects on the gut microbiota of children (Korpela et al., 2016). That separate antibiotics differentially alter the gut microbiota, suggests that subsequent microbiota-mediated effects on host health likely differ.

To what extent antibiotic treatment impacts restoration of the gut microbiota following TD is unclear. However, evidence suggesting that perturbations to the gut microbiota during TD (David et al., 2015) and antibiotic use (Dethlefsen and Relman, 2011) are followed by a subsequent remodeling of the microbiota may provide opportunities for using gut microbiota targeted interventions [e.g., probiotics or prebiotics (Ladirat et al., 2014)] to restore or favorably restructure the gut microbiota following TD. Such interventions may prove critical as evidence suggests that some antibiotic-induced perturbations to gut microbiota composition and function can persist for months to years (Jernberg et al., 2007; Jakobsson et al., 2010; Dethlefsen and Relman, 2011; Zaura et al., 2015), and that repeated antibiotic exposures may have cumulative effects (Dethlefsen and Relman, 2011). Potential consequences include the loss of critical functions within the gut microbiota, changes in resource availability and niche occupation that facilitate expansion of opportunistic pathogens, and an enrichment of anti-microbial resistance genes (Modi et al., 2014).

#### Environmental Toxicants and Pollutants

Concern over adverse health effects resulting from occupational exposures of military personnel to environmental toxicants and pollutants during training or deployment is longstanding. Moreover, future military deployments will likely occur in urban environments where risk of exposure to toxic industrial chemicals and toxic industrial materials is high (Stallings et al., 2015). These exposures may occur from a variety of sources including burn pits used to destroy solid waste, sand or soil, or from other occupational situations. For example, burn pits contain numerous mixed compounds, including PAHs, polychlorinated compounds and particulates (Masiol et al., 2016). Additionally, occupational exposures to organophosphate or carbamate pesticides occurred when these compounds were used for insect control (Sullivan et al., 2018). Cadmium, lead, arsenic and other metals have been found in contaminated soil in the numerous countries throughout the Middle East and Afghanistan (Engelbrecht et al., 2009).

Exposures to environmental toxicants have been studied mainly for long term systemic health effects on respiratory illness (Falvo et al., 2015) and cognition (Sullivan et al., 2018), among others, but there is increasing evidence that these compounds also affect the gut microbiota. For example, exposing mice to cadmium for 10 weeks altered energy metabolism and gut microbiota composition at the phyla (Firmicutes and Proteobacteria decreased, and Bacteroidetes increased) and family levels (Zhang S. et al., 2015). Concomitant increases in serum LPS concentrations were associated with an increase in Bacteroidaceae and other changes in microbiota structure. Arsenic and lead have also been shown to impact gut microbiota composition and metabolic activity (Breton et al., 2013; Lu et al., 2014). For example, arsenic exposure over 4 weeks significantly altered gut microbiota composition in mice, and compositional changes were correlated with changes in fecal and urinary metabolites including reductions in indole containing compounds, isoflavone metabolites, and bile acids (Lu et al., 2014). Interestingly, subsequent work suggested that arsenic-mediated changes in murine gut microbiota composition and functional capacity may be sex specific (Chi et al., 2016). Cadmium and lead also appear to impact the murine gut microbiota, with 8 weeks exposure to either compound having been reported to reduce diversity and relative abundance of butyrate-producing Lachnospiraceae, and increase relative abundances of Lactobacillaceae and several genera within the family Erysipelotrichaceae (Breton et al., 2013). In addition to heavy metals, polychlorinated biphenyls have been shown to alter murine gut microbiota composition by reducing the abundance of most bacteria in the community, and the pro-inflammatory phyla Proteobacteria in particular (Choi et al., 2013). Interestingly, polychlorinated biphenyls did not affect the gut microbiota in exercised mice (Choi et al., 2013). Finally, *in vitro* work using a gut model bioreactor demonstrated that 30 days exposure to the organophosphate pesticide chlorpyrifos strongly increased *Enterococcus* spp., moderately increased *Bacteroides* spp., strongly reduced *Lactobacillus*, and slightly reduced *Bifidobacterium* spp. (Joly et al., 2013). Rats gavaged with chlorpyrifos showed similar results but to a lesser extent, suggesting that chronic exposure to oral low-dose chlorpyrifos may have adverse effects on the gut microbiota (Joly et al., 2013).

Polyaromatic hydrocarbons are persistent organic compounds that can bioaccumulate in organisms, and are known environmental and food-borne contaminants (Douben, 2003). Benzo[a]pyrene (B[a]P) is a well characterized PAH compound that is mutagenic and carcinogenic in animals (Huderson et al., 2013) and a human group 1 carcinogen. While examination of B[a]P in a batch fecal fermentation showed no dose-response effect on the microbial community composition, microbial activity was altered. Specifically, microbial production of volatile organic compounds (also known as the volatolome), and the microbial metatranscriptome were altered by B[a]P in a dose-dependent manner (Defois et al., 2017). Changes to the volatolome represented a disruption to the normal microbial ecology, and metatranscriptomic changes suggested expression of adaptation mechanisms to cope with the presence of B[a]P. Altered pathways suggested downregulation of carbohydrate metabolism, and an upregulation of DNA repair and replication, and of aromatic compound, vitamin, cofactor metabolism, and cell wall compound metabolism. The same group demonstrated that 28 d oral exposure of mice to B[a]P resulted in moderate intestinal inflammation and microbial community shifts that included reductions in the relative abundance of anti-inflammatory taxa (e.g. *Lactobacillus* and *Akkermansia*), and increases in the relative abundance of several potentially pro-inflammatory taxa (e.g. *Turicibacter*) (Ribiere et al., 2016).

Particulate matter is a component of air pollution that could trigger and accelerate development of GI diseases, particularly in genetically susceptible individuals (Salim et al., 2014). This is manifested by increased GI permeability, decreased colonic motility and clearance, and altered gut microbiota composition and function. In support, exposing mice to high doses of urban PM causes oxidant-dependent GI epithelial cell death, disruption of tight junction proteins, intestinal inflammation, and increased GI permeability (Mutlu et al., 2011; Kish et al., 2013). When microbial induced colonic inflammation was modeled using IL10^-/-^ mice, long term exposure to high levels of particulates increased pro-inflammatory cytokines, altered SCFA production (increased BCFAs and reduced butyrate), increased relative abundances of Firmicutes and Verrucomicrobia, and decreased Bacteroidetes (Kish et al., 2013). Additional work is needed to determine if the observed microbiota alterations are caused directly by PM exposure, changes in the host immune response, or both.

In summary, although the specific effects differ, a growing evidence base indicates that environmental toxicants and pollutants may elicit changes in microbiota composition and metabolic activity (although not always both), changes in GI function, and, in some cases, GI inflammation. A limitation of this evidence base is that, commonly, high doses of toxic compounds are used for relatively short periods of time in small animals. In contrast, most human exposures to these compounds are at lower doses over longer periods of time. As such, to what extent findings from animal and *in vitro* studies translate to humans is unclear. Finally, it is becoming increasingly evident that the gut microbiota plays a multifaceted role regarding exposures to toxic compounds. The host microbiota (gut, skin and respiratory) represents the first interface between an exogenous chemical and the clinical disease induced by a toxicant (Dietert and Silbergeld, 2015). This interface includes microbial-modulation of the host response to toxicant exposure (Silbergeld, 2017). The microbiota is therefore not only subject to toxicant effects, but toxicants are subject to modification by the microbiota, potentially resulting in altered toxicity profiles. This suggests that the gut microbiota might be useful as an exposure surveillance tool and as a community that can be leveraged to mitigate toxicant exposures (Arcidiacono et al., 2018).

#### Noise

The high prevalence of hearing problems in military personnel and veterans (Theodoroff et al., 2015) suggests that exposure to high levels of noise may be common during military service. Aside from directly affecting the ear, exposure to this acoustic stress activates the SNS and HPA-axis, thereby eliciting a classical stress response as reflected by increased circulating concentrations of glucocorticoids and catecholamines in both animals and humans exposed to various durations and levels of noise (Ising and Kruppa, 2004; Kight and Swaddle, 2011). Thus, the deleterious effects of acoustic stress extend to other organ systems including the GI tract. In support, exposing rodents to acoustic stress has been shown to decrease expression of intestinal tight junction proteins (Cui et al., 2018), increase intestinal permeability (Bijlsma et al., 2001), alter GI motility (Gue et al., 1989; Mu et al., 2006), induce gastric ulcers (Liu et al., 1998; Mu et al., 2006), and promote inflammation and tissue damage in the intestine (Miranda and Roux, 2017). To what extent these effects impact the gut microbiota is not well characterized. However, one recent study using a mouse model of accelerated aging reported that exposure to low or high levels of noise for 4 h/days over 30 days resulted in an altered cecal microbiota, characterized primarily by an increase in the Firmicutes/Bacteroidetes ratio, concomitant to decreased expression of tight junction proteins in the colon and hippocampus, inflammation, and Alzheimer’s-like cognitive impairments (Cui et al., 2018). Germ free mice colonized with the microbiota from mice exposed to the high noise level demonstrated decreased expression of tight junction proteins, and increased hippocampal accumulation of amyloid-β, a protein implicated in Alzheimer’s disease (Cui et al., 2018). Whether the inflammatory phenotype and cognitive impairments were also transferred was not reported. Nonetheless, the findings suggest that, in mice, acoustic stress-induced changes in the gut microbiota may contribute to increased intestinal and blood brain barrier permeability and cognitive impairments. To what extent these findings translate to humans is unclear, as, to our knowledge, effects of acoustic stress on the human gut microbiota have not been examined.

### Physical Activity

There are several pathways by which physical activity may impact the gut microbiota (Clark and Mach, 2016; Monda et al., 2017). First, strenuous physical activity (≥60–70% VO_2max_), especially if prolonged, elicits a classical stress response characterized by elevated concentrations of cortisol, epinephrine, and norephinephrine which acts to reduce splanchnic and mesenteric blood flow thereby redistributing oxygen to working muscles (Qamar and Read, 1987; van Wijck et al., 2012). The consequent reduced blood supply to the intestinal epithelium and subsequent reperfusion can cause hypoxia, acidosis, ATP depletion, free radical formation, and oxidative/nitrosative stress which collectively damage the gut barrier resulting in increased intestinal permeability (Lambert, 2008; van Wijck et al., 2012). The subsequent combination of LPS/endotoxin translocation into circulation and an undersupply of blood, nutrients, water and oxygen to the intestines, promote inflammation and GI distress such as nausea, cramping, vomiting, and diarrhea (Lamprecht and Frauwallner, 2012). Importantly, these effects are generally not observed at lower activity intensities. Further, regular moderate physical activity (i.e., exercise training) elicits physiological adaptations that act to maintain intestinal blood flow during activity and reduce inflammation (Lambert, 2008) thereby attenuating physical activity-induced gut dysfunction (Luo et al., 2014). Regular physical activity has also been shown to modulate GI motility (Oettle, 1991) which is associated with gut microbiota composition (Roager et al., 2016). Finally, regular physical activity modulates immune function with improvements seen with regular moderate exercise (Walsh et al., 2011), but immunosuppression when recovery is insufficient (Schwellnus et al., 2016). Thus, any effects of physical activity on the gut microbiota and their persistence may vary with the novelty, frequency, intensity, and duration of activity.

Several recent reviews have comprehensively characterized the effects of exercise training on murine gut microbiota composition (Cerda et al., 2016; Clark and Mach, 2016; Mach and Fuster-Botella, 2017). Despite differences in the types of animals studied, diets, duration of training (6 days – 12 weeks), intensity and duration of exercise bouts, and whether exercise was voluntary or forced, a commonality across several (Choi et al., 2013; Queipo-Ortuno et al., 2013; Petriz et al., 2014; Allen et al., 2015; Lambert et al., 2015), but not all (Evans et al., 2014; Kang et al., 2014), of the studies reviewed was an increased abundance of *Lactobacillus* and *Bifidobacterium* following exercise training. Separate studies also reported reduced abundance of the potentially pathogenic taxa Turicibacteraceae and *Turicibacter* (a genus within the Turicibacteraceae family) following voluntary exercise over 6–12 weeks (Evans et al., 2014; Allen et al., 2015). However, those observations have not been consistently reproduced in subsequent studies (Mika et al., 2015; Campbell et al., 2016; Denou et al., 2016; Welly et al., 2016; Batacan et al., 2017; Lamoureux et al., 2017). Similarly, several studies have reported increased abundances of butyrate-producing taxa (Matsumoto et al., 2008; Queipo-Ortuno et al., 2013; Campbell et al., 2016; Batacan et al., 2017; Allen et al., 2018a) and cecal butyrate concentrations (Matsumoto et al., 2008) with exercise training, while other studies have not. In a notable recent study, Allen et al. (2018a) documented increased abundances of *Akkermansia* and of an unclassified genus within the family Lachnospiraceae (which contains several butyrate-producing genera), and an increased cecal butyrate:acetate ratio in exercised mice relative to sedentary controls. Colonization of germ free mice with the gut microbiota of exercised mice induced several beneficial effects relative to mice colonized with the gut microbiota from sedentary controls, including a more favorable inflammatory profile, improved gut morphology, and an attenuated response to experimentally induced colitis (Allen et al., 2018a). To our knowledge, this study was the first to demonstrate a causal role of physical activity-induced changes in the gut microbiota in producing health benefits. Similar study designs will be informative moving forward given that physical activity does appear to influence murine gut microbiota composition, but not in any clear, consistent manner. This inconsistency is perhaps not surprising in lieu of heterogeneous study designs, and recent evidence suggesting that the effects of exercise on the murine gut microbiota may vary based on the anatomical GI region examined (Denou et al., 2016), whether exercise is voluntary or forced (Allen et al., 2015), age (Mika et al., 2015), exercise intensity (Denou et al., 2016), energy balance status (Queipo-Ortuno et al., 2013), and diet composition (Batacan et al., 2017).

To our knowledge, only one study has longitudinally examined the effects of physical activity on the human gut microbiota. In that trial, 3 days/weeks of moderate intensity exercise over 6 weeks was shown to differentially impact gut microbiota composition and fecal SCFA content of previously sedentary lean and obese adults (Allen et al., 2018b). Specifically, *Bacteroides* and *Collinsella* were increased and *Faecalibacterium* were decreased following training in volunteers with obesity while *Bacteroides* were decreased and *Faecalibacterium* and *Lachnospira* were increased following training in lean volunteers. Most of the observed changes reverted toward pre-training values during a subsequent return to sedentary behavior. Additionally, fecal SCFA concentrations and the abundance of bacterial genes involved in SCFA-production in feces were increased following training in lean but not obese volunteers (Allen et al., 2018b). Although the study design precluded definitively attributing observed effects to the exercise intervention, the data are the first to associate increases in physical activity with changes in gut microbiota composition and activity in humans.

In summary, physiologic responses to physical activity range along a spectrum of beneficial to potentially harmful which varies with the novelty, frequency, intensity, and duration of activity. That physical activity alters gut microbiota composition and function, perhaps favorably, is supported by a rapidly growing collection of rodent studies. However, findings have been inconsistent and likely vary with the novelty, frequency, intensity and duration of activity. The effect of physical activity on the human gut microbiota remains largely unexplored.

### Diet

#### Food Restriction

Diet is a predominant factor influencing gut microbiota composition and activity. Both species abundances and metabolic outputs of the human gut microbiota respond within days to changes in diet (Walker et al., 2011; Wu et al., 2011; David et al., 2014), an effect that is thought to occur through multiple interrelated pathways (Read and Holmes, 2017). The most direct pathway underpinning diet-gut microbiota interactions is the delivery of undigested macro- and micro-nutrients from the diet to the colon. Up to ∼70 g of undigested carbohydrate (Topping et al., 2003; Scott et al., 2008; Bird et al., 2010), ∼25 g of diet-derived and endogenous proteins and peptides (Bird et al., 2010; Macfarlane and Macfarlane, 2012; Yao et al., 2016), vitamins, minerals, and other unabsorbed dietary components reach the colon daily where they provide essential energy and nutrients for a variety of microbes. Other diet-microbiota interactions are less direct. Nutrient intakes influence GI physiology (e.g., transit time, pH, permeability and morphology, mucin secretion), secretion of digestive compounds (e.g., bile, enzymes), eating behavior, intestinal inflammation and oxidative stress, and host immune and nervous system function. At the extreme, completely depriving animals of food causes gut mucosa atrophy and hypoplasia, gut inflammation, decreased gut barrier integrity, increased GI permeability, and, subsequently, translocation of bacterial components into circulation resulting in systemic inflammation (for review Demehri et al., 2015; Genton et al., 2015). As such, dietary habits that deprive the gut microbiota of required or preferred substrates by altering nutrient availability and/or that include consumption of compounds which create an inhospitable environment in the gut may constitute a “stress” on a healthy gut microbiota.

Emerging evidence indicates that changes in gut microbiota composition may contribute to decrements in gut health during food restriction. Animal models consistently report altered gut microbiota composition during acute (1–3 days) (Tannock and Savage, 1974; Deitch et al., 1987; Crawford et al., 2009; Sonoyama et al., 2009; Costello et al., 2010; Kohl et al., 2014) and prolonged (e.g., hibernation) (Sonoyama et al., 2009; Carey et al., 2013; Dewar et al., 2014; Stevenson et al., 2014) fasting. Although taxonomic changes are not consistent across studies, and may be specific to the animal studied (Kohl et al., 2014), the most consistent finding appears to be that of an increased abundance of taxa capable of degrading host-derived mucosal glycans such as Bacteroidetes and *Akkermansia* during fasting (Crawford et al., 2009; Sonoyama et al., 2009; Costello et al., 2010; Kohl et al., 2014). Similarly, murine and porcine studies using total parenteral nutrition, a model whereby complete nutrition is provided intravenously to bypass the gut, have demonstrated decreased gut microbiota diversity (Harvey et al., 2006; Miyasaka et al., 2013), and increased abundances of mucolytic (e.g., *Akkermansia* and Bacteroidetes) and pro-inflammatory (e.g., Proteobacteria and Enterobacteriaceae) taxa in animals receiving total parenteral nutrition (Deplancke et al., 2002; Harvey et al., 2006; Hodin et al., 2012; Miyasaka et al., 2013). Sulfates released during degradation of the heavily sulfated mucins within the mucus layer may help facilitate the growth of pro-inflammatory sulfate-reducing bacteria (e.g., *Desulfovibrio*) (Gibson et al., 1993). Moreover, pro-inflammatory Proteobacteria are thought to be more resistant to nutrient-depleted conditions than other commensal microbes (Demehri et al., 2015). Taken together, these observations suggest that the absence of diet-derived substrate stresses the gut microbiota by requiring microbes to rely on host factors to survive. This results in the growth of bacteria capable of metabolizing host-derived mucosal glycans and pro-inflammatory taxa which may contribute to mucus barrier degradation, increased GI permeability, and subsequent inflammation.

In healthy humans, complete food deprivation for extended periods is uncommon. However, interrelationships between food restriction and the gut microbiota are increasingly being studied in the context of treatments for overweight and obesity. A recent meta-analysis of those studies (*n* = 11 trials) demonstrated that weight loss diets were associated with reduced total bacteria abundance (3 of 5 trials), reduced abundance of butyrate-producing bacteria (6 of 10 trials), and a trend for increased abundances of *Lactobacillus, Akkermansia muciniphila*, and *Faecalibacterium prausnitzii* (5 of 10 trials), with equivocal results for diversity (increased in 2 of 4 trials) and phyla-level abundances (Seganfredo et al., 2017). However, differences in the macronutrient compositions of the diets were noted to influence some of the results which precludes separating effects of food restriction from those attributable to changes in diet composition.

Although manipulating food intake without altering dietary macronutrient composition has been shown to impact gut microbiota composition in healthy adults (Jumpertz et al., 2011; Faith et al., 2013), it is likely a combination of both the quantity and proportion of different nutrients consumed in the diet that ultimately impact the gut microbiota. Below we consider evidence that diet composition, or, alternately, the insufficiency or excess of certain nutrients, can stress a healthy gut microbiota. Of note, the use of diet and individual nutrients to favorably modulate the gut microbiota is beyond the scope of this review. We acknowledge that certain non-digestible carbohydrates (NDC; e.g., galacto-oligosaccharides, fructo-oligosaccharides) have established beneficial effects on the gut microbiota (Gibson et al., 2017), and that other NDC types (Rastall, 2010), nutrients such as plant polyphenols (Duenas et al., 2015), and mixed NDC-polyphenol sources such as whole grains (Costabile et al., 2008; Martinez et al., 2013; Vanegas et al., 2017) are emerging as beneficial modulators of the gut microbiota. For readers interested in the full spectrum of diet-gut microbiota-host interactions we suggest several excellent reviews on the topic (Ha et al., 2014; Salonen and de Vos, 2014; Louis et al., 2016; Sonnenburg and Backhed, 2016; Yao et al., 2016; Espin et al., 2017; Read and Holmes, 2017).

#### Diet Composition

Within the varied diets of most healthy humans, NDC provide the primary carbon source for many gut microbes. NDC-containing foods often include multiple NDC types that are diverse in structure, composition, degree of polymerization, and in the types of glycosidic bonds within the polymer. The myriad enzymes required to metabolize this diversity are contained not within individual species of the gut microbiota, but within the collective genome of the gut microbiota (Martens et al., 2014). Several gut bacteria taxa, including *Bacteroides*, multiple Firmicutes (e.g., *Roseburia, Eubacterium, Clostridium, Lactobacillus*, and *Ruminococcus*), and *Bifidobacterium* degrade NDC into smaller polymers which can then be metabolized by cross-feeding saccharolytic microbes (Koropatkin et al., 2012). While some bacteria possess multiple systems for degrading and metabolizing NDC, others have a more selective capacity (Koropatkin et al., 2012; White et al., 2014). Consequently, the availability of different carbohydrate types can differentially promote the growth of gut microbes (Sonnenburg and Sonnenburg, 2014; Sonnenburg and Backhed, 2016). Cross-feeding on breakdown products and metabolic intermediates amongst gut microbes further contributes to gut microbiota diversity and metabolism, and the byproducts of this metabolism (e.g., SCFA) generally create a hospitable environment for beneficial microbes, by reducing the luminal pH for example (Flint, 2012). Consequently, low NDC intakes may stress the gut microbiota by reducing the availability of preferred or required substrates, and by unfavorably altering the colonic environment.

Similar to carbohydrates, proteins provide fermentative substrate to the gut microbiota. The primary proteolytic species in the human gut belong to the genera *Bacteroides* and *Clostridium* (Roberfroid et al., 2010). However, the requirement for amino acids and nitrogen among gut microbes is ubiquitous, with microbes using diet- and host-derived amino acids and nitrogen for protein synthesis (Lin et al., 2017). As such, reducing the amount of proteins, peptides and amino acids reaching the colon could stress the microbiota. Conversely, *in vitro* studies suggest that some metabolites of protein fermentation may be toxic to intestinal cells and increase paracellular permeability (Verbeke et al., 2015; Yao et al., 2016). If extant *in vivo*, these effects could also stress the gut microbiota by unfavorably modulating host physiology.

Unlike carbohydrate and protein, the primary effects of dietary fat on the gut microbiota are thought to be more indirect and mediated by bile acid secretion, and through modulation of GI inflammation and barrier integrity (Shen et al., 2014; Wahlstrom et al., 2016). Bile acids have antimicrobial effects against some gut commensals (Lorenzo-Zuniga et al., 2003), but may also enrich for bile-acid metabolizing bacteria such as the potentially harmful sulfate-reducing genus *Bilophila* (family Desulfovibrionaceae) (Devkota et al., 2012; Caesar et al., 2015). Additionally, bile acids, which are conjugated in the liver, are deconjugated by gut bacteria. High levels of conjugated relative to deconjugated bile acids may stress the microbiota and promote dysbiosis by increasing GI permeability (Tran et al., 2015).

Animal models have consistently demonstrated changes in gut microbiota composition in response to high fat diets (≥40% total energy intake) which can generally, but not exclusively, be considered unfavorable (e.g., decreased *Bifidobacterium*, and increased sulfate-reducing and pro-inflammatory taxa) (Ha et al., 2014; Shen et al., 2014). In several studies, those changes have occurred concomitant to decreased gut barrier integrity, increased GI permeability, endotoxin translocation, and low-level inflammation which contribute to metabolic dysfunction, and induction and maintenance of obesity (Cani et al., 2008; Turnbaugh et al., 2008; Hildebrandt et al., 2009; de La Serre et al., 2010; Lam et al., 2015). However, the capacity of high fat intake to stress the microbiota depends on fatty acid composition due to differential effects on fatty acid type on bile acid secretion and inflammation (Devkota et al., 2012; Ghosh et al., 2013; Huang et al., 2013; Kaliannan et al., 2015). Further, the high fat diets (often referred to as “Western” diets) used in these studies are commonly low in NDC and are often compared to NDC-rich standard chow diets. Thus, the combination of a high fat intake and low NDC intake, rather than fat alone, likely constitutes the true stress on the gut microbiota in these studies, with the magnitude of stress being modulated by the types of fatty acids consumed.

Animal studies using multiple simplified diets comprised of different combinations of individual macronutrient sources (*n* = 17–25 diets) have reported that protein and/or carbohydrate rather than fat drive effects of dietary macronutrient intake on gut microbiota composition (Faith et al., 2011; Holmes et al., 2017). Holmes et al. (2017) attributed this relationship to changes in nutrient availability in the colon which in turn determines intestinal nitrogen availability. Specifically, low protein and carbohydrate intakes reduced diet-derived nitrogen availability thereby favoring microbes that utilize host nitrogen sources (e.g., proteoglycans such as mucin). This “limitation-type” response was associated with higher compositional diversity, increased abundance of taxa capable of metabolizing host proteoglycans (e.g., *Akkermansia, Ruminococcus, Bacteroidetes*) and producing butyrate (*Eubacterium, Butyvibrio*), and a healthier host phenotype (Holmes et al., 2017). Reducing NDC intake to a greater extent than was done in that study or completely eliminating NDC from animal diets similarly increased abundance of mucus-degrading taxa and expression of genes targeting mucus catabolism, but also reduced community diversity by decreasing the abundance of, and possibly eliminating, multiple taxa lacking the enzymatic machinery to degrade host mucins (Desai et al., 2016; Sonnenburg et al., 2016). These decrements may reduce the thickness of the colonic mucus layer, thereby allowing bacteria to gain closer proximity to the intestinal epithelium and enhancing susceptibility to enteric pathogens (Desai et al., 2016; Schroeder et al., 2018).

Low NDC intakes may also stress the human gut microbiota, particularly when coupled with high fat, and possibly high protein intakes. In support, reducing carbohydrate and NDC intakes while increasing fat intake reproducibly reduced the abundance of *Roseburia–Eubacterium rectale* and fecal butyrate concentrations in adults consuming hypocaloric higher protein (∼30% total energy) diets (Duncan et al., 2007, 2008; Russell et al., 2011) (**Table 3**). This effect was likely driven by reduced NDC intake, and lower starch intake in particular, as these beneficial butyrate-producing taxa metabolize mono- and polysaccharides (Desai et al., 2016) and are enriched following increased resistant starch intake (Abell et al., 2008; Martinez et al., 2010; Walker et al., 2011). Notably, a recent meta-analysis reported that reductions in *Roseburia* and *Eubacterium rectale*, and *Bifidobacterium* as well, are consistently documented in longitudinal analyses of studies examining low carbohydrate, high protein, energy restricted diets (Seganfredo et al., 2017). In contrast, maintaining NDC and fat intakes while reducing carbohydrate and increasing protein intakes, had no impact on the relative abundances of *Roseburia, Eubacterium rectale* or any other taxa in another study, although fecal butyrate concentrations were reduced (Beaumont et al., 2017). In other studies, reduced carbohydrate intake combined with increased protein and fat intakes did not affect *Roseburia* or *Eubacterium rectale*, but rather reduced *Bifidobacterium* abundance (Brinkworth et al., 2009; Fava et al., 2013) and fecal butyrate (Brinkworth et al., 2009), or had no impact on gut microbiota composition compared to control diets (Wu et al., 2011; Windey et al., 2012).

**Table 3:** Randomized clinical trials examining effects of diet macronutrient or energy manipulation on human gut microbiota composition and metabolites.

Reference	Design^1^	Microbiota method	Results- Microbiota	Results- Microbiota metabolites
**Carbohydrate and fat manipulation**				
Duncan et al., 2007	*n* = 18 obese M, 4 weeks, CO; hypocaloric high PRO diets: highPRO + modCHO (35/30/35, 12g NSP), and highPRO + lowCHO (4/30/66, 6 g NSP)	Targeted; FISH	lowCHO vs modCHO: ↓*Roseburia–E. rectale* group; *↔*Total bacteria, *Bacteroides–Prevotella, F. prausnitzii, Bifidobacterium*, Clostridial clusters XIVa, XIVb, IX, *R. bromii, R. flavefaciens, Lactobacillus-Enterococcus*	lowCHO vs. modCHO: ↓Fecal butyrate; ↔fecal acetate, propionate, BCFA, NH_3_
			Both diets: ↓Total bacteria, *Bifidobacterium*, *Roseburia–E. rectale* group	Both diets: ↓Fecal acetate, propionate, butyrate, isovalerate, valerate, NH_3_
Duncan et al., 2008	*n* = 23 obese M, 4 weeks, CO; hypocaloric high PRO diets: highPRO + modCHO (35/30/35, 12 g NSP), and highPRO + lowCHO (4/30/66, 6 g NSP)	Targeted; FISH	lowCHO vs. modCHO: ↓*Roseburia–E. rectale* group; *↔Bacteroides, Firmicutes, Bifidobacterium, Clostridium coccoides*	NA
			Both diets: ↓total bacteria, *Bifidobacterium*, *Roseburia–E. rectale* group; ↑*C. coccoides*	
Russell et al., 2011	*n* = 17 obese M, 4 weeks, CO; hypocaloric high PRO diets: highPRO + modCHO (35/28/37, 13 g NSP), and highPRO + lowCHO (5/29/66, 9 g NSP)	Targeted; FISH	lowCHO vs. modCHO: ↓*Roseburia–E. rectale* group; *↔Bacteroides*, Lachnospiraceae, *F. prausnitzii*	lowCHO vs. modCHO: ↓Fecal acetate, butyrate, total SCFA, plant-derived phenolics, fatty acid-derived bacterial metabolites; ↑Fecal pH, N-nitroso compounds
			Both diets: ↓total bacteria	Both diets: ↑Fecal isovalerate, valerate, *N*-nitroso compounds; ↓fecal propionate
**Carbohydrate and protein manipulation**				
Beaumont et al., 2017	*n* = 38 (13M) ovwt, 3 weeks, PA; PRO and CHO supplementation: casein (35/34/29, 25 g fiber), soy PRO (37/32/29, 22 g fiber), or CHO (54/14/29, 20 g fiber)	16S rRNA gene sequencing	No effects of diet on fecal or rectal mucosa microbiota	Fecal metabolome: ↓butyrate and ↑AA-derived bacterial metabolites (e.g., BCFA) which differed by PRO group
				Urine metabolome: ↑AA-derived bacterial metabolites (e.g., BCFA) which differed by PRO group
				Plasma metabolome: No differences between groups in bacterially derived metabolites
**Carbohydrate, protein and fat manipulation**				
Ley et al., 2006	*n* = 12 obese adults, 1 year, PA; hypocaloric diets: modFat (30% kcal fat) or lowCHO (25% kcal CHO)	16S rRNA gene sequencing	No differences between groups	NA
			Both groups: ↑Bacteroidete*s*; ↓Firmicutes	
Brinkworth et al., 2009	*n* = 91 (36M) ovwt/obese, 8 weeks, PA; lowCHO + highFAT (5/35/60, 13 g fiber) or highCHO + lowFAT (46/24/30, 32 g fiber)	Targeted; culture	lowCHO vs. high CHO: ↓Total anaerobes, *Bifidobacterium*; ↔Total anaerobes, coliforms, *Escherichia coli, Lactobacillus*	lowCHO vs. high CHO: ↓Fecal acetate, butyrate, total SCFA; ↔fecal pH and NH_3_, urinary phenols and *p*-cresol
Wu et al., 2011	*n* = 10 (6M) healthy adults, 10 days, PA: highFiber + lowFAT (69/18/13, 52 g fiber); lowFiber + highFAT (35/27/38, 22 g fiber)	16S rRNA gene sequencing; SM	Shifts in composition within 24 h, but no differences between diets.	NA
Windey et al., 2012	*n* = 20 (6M) adults, 2 weeks, CO: lowPRO (60/12/17, 17 g fiber); highPRO (41/27/32, 15 g fiber)	DGGE	No differences	highPRO vs lowPRO: ↑Urinary *p*-cresol, fecal isobutyric and isovaleric acids; ↔fecal *p*-cresol, acetate, propionate, butyrate
Fava et al., 2013	*n* = 88 (43M) adults at risk for MetS, 24 weeks, PA: highSFA + highGI (43/15/38) highSFA + highGI (43/15/38) highMUFA + highGI (43/16/38) highMUFA + lowGI (46/17/35) highCHO + highGI (51/20/27) highCHO + lowGI (55/18/23) All diets 17–22 g NSP	Targeted; FISH	highMUFA: ↓Total bacteria	↔Acetate, butyrate, propionate, valerate, caproate
			highCHO + highGI: ↑*Bifidobacterium* vs. highSFA + highGI; ↑*Bacteroides* vs. baseline	
			highCHO: ↑*Bifidobacterium* vs. baseline	
			highCHO + lowGI: ↑*F. prausnitzii* vs. baseline	
David et al., 2014	*n* = 10 (6M) adults, 5 days, CO; Plant-based diet (68/10/22, 26 g fiber/1000 kcal), and Animal-based diet (0/30/70, 0 g fiber)	16S rRNA gene sequencing	Animal diet: Transient change in diversity, changes in 22 bacterial clusters, ↑bile acid tolerant and putrefactive taxa (e.g., *B. wadsworthia, Alistipes putredinis, Bacteroides*), *Akkermansia, R. gnavus, Escherichia*; ↓*Roseburia, E. rectale, R. bromii*	Animal vs plant-metabolites: ↓Fecal acetate, butyrate; ↑fecal isovalerate, isobutyrate, deoxycholic acid (secondary bile acid)
			Plant diet: Changes in 3 clusters, ↓*B. wadsworthia, Clostridium, Ruminococcus*	Animal vs. plant-gene expression: ↑bile salt hydrolases, sulfite reductases, AA catabolism; ↓AA biosynthesis
**Energy balance manipulation**				
Jumpertz et al., 2011	*n* = 21M (lean or obese), 3 days, CO; energy manipulation: 2400 kcal/day (60/24/16), and 3400 kcal/day (60/24/16); diets were fiber-matched	16S rRNA gene sequencing	Overeating: ↓*Bacteroidales*; ↑*Clostridia* Undereating: ↑*Bacteroidales*; ↓*Clostridia*	NA


*AA, amino acid; BCFA, branched-chain fatty acid; CHO, carbohydrate; CO, crossover study; DGGE, differential gradient gel electrophoresis; FISH, fluorescence in-situ hybridization; GI, glycemic index; MetS, metabolic syndrome; MUFA, monounsaturated fatty acid; NSP, non-starch polysaccharides; ovwt; overweight; PA, parallel-arm randomized trial; PRO, protein; SCFA, short-chain fatty acid; SFA, saturated fatty acid; SM, shotgun metagenomics; ↑, increase/higher; ↓, decrease/lower; ↔, no change/no difference. Genus abbreviations: *B., Bilophila; C., Clostridium; E., Eubacterium; F; Faecalibacterium; R, Ruminococcus*. ^*1*^Macronutrient proportions are percent total energy from CHO/PRO/FAT.*

Extreme dietary shifts appear to have more robust effects on the human gut microbiota. In support, David et al. (2014) compared changes in the fecal microbiota of adults fed a high-fiber plant-based diet or a low carbohydrate, fiber-free, high-protein, high-fat animal-based diet. Effects of the animal-based diet included increased abundances of bile-tolerant taxa (*Alistipes, Bilophila, Bacteroides*) in association with increased bile acid concentrations, increased abundances of putrefactive taxa (i.e., *Alistipes putredinis* and *Bacteroides* spp.) in association with increased fecal BCFA concentrations, and decreased abundances of saccharolytic taxa (e.g., *Roseburia, Eubacterium rectale, Ruminococcus bromii, Faecalibacterium prausnitizii*) in association with reduced SCFA concentrations (David et al., 2014). Transcriptomic analyses indicated that the animal based diet increased bacterial expression of genes involved in amino acid catabolism and bile acid deconjugation, thereby demonstrating functional adaptations to changes in the colonic environment (David et al., 2014). Finally, in an interesting non-randomized trial, O’Keefe et al. (2015) reported that feeding rural Africans a low-fiber, high-fat “Western” diet in place of their habitual high-fiber, low-fat “African-style” diet was associated with reduced concentrations of fecal butyrate, acetate, propionate, and BCFA, increased fecal concentrations of deconjugated bile acids, and intestinal inflammation (O’Keefe et al., 2015). Opposite effects were generally observed in African Americans who were fed the high-fiber, low-fat rural African-style diet in place of their habitual Western-style diet (O’Keefe et al., 2015). Taken together, these studies provide evidence that the human gut microbiota may be “stressed” by low NDC, high protein, high fat diets, but that effects may be somewhat subtle in the absence of substantial dietary shifts. However, macronutrient sources also matter. For example, reducing intake of fermentable saccharides in the absence of differences in dietary fiber, resistant starch, fat, carbohydrate or protein intakes has been shown to increase fecal pH and reduce fecal counts of *Lactobacilllus*, *Bifidobacterium*, and several butyrate-producing taxa (Halmos et al., 2015). Similarly, although largely untested in randomized clinical trials, *in vitro* and animal studies suggest protein source and fatty acid type may likewise differentially alter gut microbiota composition and activity (Singh et al., 2017).

Diet-mediated changes in microbiota composition and function cannot always be solely attributed to altered dietary macronutrient composition because micronutrient intakes are often also altered. In support, growing evidence indicates that plant polyphenols favorably modulate gut microbiota composition and metabolic activity (Duenas et al., 2015; Espin et al., 2017) which suggests that removing these substrates from the diet could unfavorably modulate the gut microbiota independent of macronutrient intake. Further, all bacteria have micronutrient requirements, and compete for and utilize diet-derived vitamins and minerals to help meet those requirements (Degnan et al., 2014; Biesalski, 2016; Hibberd et al., 2017). Many vitamins and minerals also have essential roles in enterocyte health, and gut barrier and immune function indicating that deficiencies or excess may indirectly impact the gut microbiota. In support, dysbiosis has been implicated as both a cause and consequence of undernutrition-related health sequelae (Smith et al., 2013; Blanton et al., 2016). However, relative to macronutrients, the effects of micronutrient insufficiencies or excess on the gut microbiota are understudied (Mach and Clark, 2017). Many bacteria are capable of synthesizing various vitamins (Biesalski, 2016) which could help mitigate any direct “stress” of most dietary vitamin deficiencies on the microbiota. In contrast, mineral requirements must be met through exogenous sources. That mineral requirements differ across taxa indicates mineral availability could differentially stress the gut microbiota by selecting for potentially harmful microbes or depriving beneficial microbes. For example, several beneficial microbes (e.g., *Bifidobacterium*) generally have low iron requirements whereas the growth and virulence of several pro-inflammatory (e.g., Enterobacteriaceae) and pathogenic microbes (e.g., *Salmonella*) are enhanced by iron (Kortman et al., 2014).

Iron and zinc are perhaps the best studied minerals with respect to how variations in mineral intakes impact the gut microbiota. *In vitro* and animal studies have reported that low iron or zinc availability may unfavorably alter the gut microbiota and decrease SCFA production, although in some studies favorable effects or no effects have also been reported (Tompkins et al., 2001; Dostal et al., 2012, 2013, 2014, 2015; Kortman et al., 2014; Reed et al., 2015; Mayneris-Perxachs et al., 2016; Zackular et al., 2016; Hibberd et al., 2017). At the other end of the spectrum, oral iron supplementation has been shown to enrich for pro-inflammatory (e.g., Enterobacteriaceae) and pathogenic (e.g., *Salmonella*) taxa, deplete beneficial taxa (e.g., *Lactobaciulls, Roseburia, Eubacterium rectale*), increase diarrhea incidence, and/or increase fecal markers of inflammation in murine models and in several studies of undernourished infants (Zimmermann et al., 2010; Werner et al., 2011; Kortman et al., 2014; Jaeggi et al., 2015; Paganini et al., 2017), particularly in environments where the pathogen burden is high (Paganini and Zimmermann, 2017). Similarly, zinc supplementation was recently shown to reduce diversity of the murine gut microbiota, and increase susceptibility to the enteropathogen *Clostridium difficile* (Zackular et al., 2016), although other studies suggest decreased virulence of other enteropathogens with zinc supplementation (Crane et al., 2011; Mellies et al., 2012). Iron deficiency is prevalent in some groups of military personnel (McClung et al., 2009a,b; Karl et al., 2010), which has been attributed in part to suboptimal intakes, but also to inflammation which reduces absorption of dietary iron (Gaffney-Stomberg and McClung, 2012), and possibly also zinc (Hennigar and McClung, 2016). To what extent changes in the luminal availability of iron, zinc or other minerals due to fluctuations in intake or inflammation impact the human gut microbiota is undetermined.

In summary, changes in the absolute and relative amounts of nutrients consumed in the diet alters host physiology, and nutrient availability and environmental conditions in the colon. Animal studies have demonstrated that both total food deprivation and low NDC intakes stress the gut microbiota initiating an adaptive response characterized by an increased abundance of mucolytic and, in some cases, pro-inflammatory taxa, an increased abundance and expression of genes involved in mucus degradation (e.g., mucin), a reduced abundance of beneficial butyrate-producing taxa, and a reduced diversity and expression of genes encoding carbohydrate degrading enzymes (Desai et al., 2016; Sonnenburg et al., 2016). When low-NDC intakes are paired with high-fat and high-protein intakes, adaptive responses may also include increased abundance and expression of genes required to metabolize newly available nutrients (e.g., amino acids) and related compounds (e.g., bile acids) (Faith et al., 2011; Devkota and Chang, 2013). Collectively, these effects may promote inflammation, impair gut barrier function, and increase GI permeability.

Similar adaptive responses have also been reported in a limited number of human studies although effects may be somewhat more subtle than reported in animals absent of substantial changes in diet. As such, to what extent different dietary macro- and micro-nutrients “stress” the gut microbiota when consumed in excess or in inadequate amounts is unresolved. Finding an answer to the question is difficult in part because nutrients are not consumed in isolation. Both the amounts and proportions of macro- and micro-nutrients in the diet, as well as factors impacting nutrient digestibility and bioavailability (e.g., food processing/cooking, nutrient-nutrient interactions, host physiology, gut microbiota composition) will determine the ultimate impact of diet on the gut microbiota. Additionally, it is worth noting that recent studies have suggested that non-nutritive dietary components such as the artificial sweetener saccharin (Suez et al., 2014), and the emulsifiers carboxymethylcellulose and polysorbate-80 (Chassaing et al., 2015, 2017) unfavorably impact murine physiology by modulating the gut microbiota. As such, elucidating how dietary patterns may stress a healthy gut microbiota is an exceedingly complex endeavor that will require combinations of *in vitro, ex vivo* and clinical studies to unravel.

## Conclusion

**FIGURE 1:**
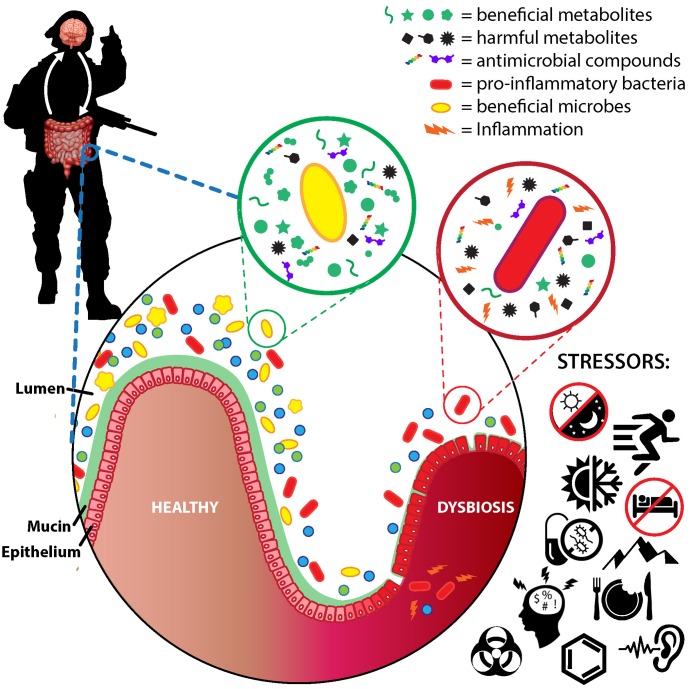
Military-relevant stressors and the gut microbiota. Military personnel can be exposed to extremes and combinations of psychological, environmental (e.g., altitude, heat, cold, and noise) and physical (e.g., physical activity, sleep deprivation, and circadian disruption) stressors. These stressors induce central stress responses that ultimately alter gastrointestinal and immune function which may lead to changes in gut microbiota composition, function and metabolic activity. Other stressors such as diet, enteric pathogens, environmental toxicants and pollutants, and antibiotics can alter gut microbiota composition and activity through direct effects on the gut microbiota, and indirectly through effects on gastrointestinal and immune function. Stress-induced changes in the gastrointestinal environment may elicit unfavorable changes in gut microbiota composition, function and metabolic activity resulting in a dysbiosis that further compromises gastrointestinal function, and facilitates translocation of gut microbes and their metabolites into circulation. Alternately, evidence suggests that some stressors (e.g., healthy diet, cold, and physical activity) may favorably modulate the gut microbiota. To what extent these changes impact the health, and physical and cognitive performance of military personnel is currently unknown.

There is increasing recognition that humans are ‘superorganisms’ or ‘holobionts’ comprised of an integrated network of human cells and microorganisms whose dynamic bidirectional interactions react and respond to environmental pressures to influence health (Gilbert et al., 2012). The gut microbiota, comprising the densest microbial community within this superorganism, demonstrates resilience to perturbation and long-term stability (Lozupone et al., 2012; Faith et al., 2013). However, preclinical studies clearly demonstrate that gut microbiota composition and activity is malleable over shorter time frames, and influenced by psychological, physical, and environmental stressors (**Figure 1**). Further, these studies demonstrate that the gut microbiota’s response to stress over both the short- and long-term, can potentially be both health promoting (e.g., with cold exposure), health-degrading (e.g., with psychological stress, circadian disruption, and high altitude), or both (e.g., with physical activity and diet). The implication is that the gut microbiota can be a factor contributing to adverse stress-associated health outcomes, but may also provide a tool for favorably modulating the host stress response.

Military personnel frequently operate in austere environments in which they are exposed to a variety of stressors that challenge health, cognition, and physical function. Transient health decrements associated with exposure to these stressors [e.g., musculoskeletal injury (Jacobs et al., 2014), immunosuppression (Institute of Medicine, 1999), inflammation (McClung et al., 2013; Pasiakos et al., 2016), illness and infection (Connor et al., 2012; Sanchez et al., 2015), and cognitive and psychological impairments (Hoge et al., 2004; Lieberman et al., 2005)] are interrelated and have multi-factorial etiologies, but could be associated with the gut microbiota given the varied roles of this community in modulating nutrient metabolism (Holmes et al., 2012; Weaver, 2015), GI permeability and inflammation (Cani et al., 2012; Wells et al., 2017), immunity (Hooper et al., 2012), and the gut-brain axis (Cryan and Dinan, 2012; Foster and McVey Neufeld, 2013; Forsythe et al., 2016). Further, while active duty military personnel generally report equivalent or better overall physical health compared to civilian counterparts (Hoerster et al., 2012; Lehavot et al., 2012), military veterans report higher rates of mental health disorders, cardiovascular diseases, arthritis, cancer, and obesity relative to civilians (Hoerster et al., 2012; Lehavot et al., 2012; Breland et al., 2017). Notably, the gut microbiota has been associated with all of those conditions (Turnbaugh et al., 2009; Kostic et al., 2012; Tremaroli and Backhed, 2012; Koeth et al., 2013; Scher et al., 2013; Luna and Foster, 2015; Leclercq et al., 2016; O’Keefe, 2016). The gut microbiota could therefore be an underappreciated mediator of health outcomes resulting from exposure to military stressors. However, as reviewed above, there is a general lack of human studies which have longitudinally followed or experimentally manipulated exposures to stress while examining changes in the gut microbiota and relevant outcomes, or examined how the gut microbiota may influence responses to stress. Although animal, *in vitro*, *ex vivo*, and *in silico* investigations are invaluable for gaining insight into host–microbiota dynamics, all models have their limitations, and it cannot be assumed that findings translate to humans (Nguyen et al., 2015). Likewise, cross-sectional studies conducted in humans (which were generally not reviewed herein) are useful for generating hypotheses, but warrant cautious interpretation given the myriad potential confounding factors that can impact host-microbiota associations. Therefore, to what extent transient or cumulative exposures to psychological, environmental and physical stressors, especially when experienced in combination, meaningfully impact the gut microbiota of military personnel is presently unclear.

Nonetheless, the provocative preclinical evidence reviewed highlights a need for translational research aiming to elucidate the impact of psychological, environmental and physical stressors on the human gut microbiota, and the associated health implications. This work will transcend military applications given the increasing exposure of many civilian populations to similar stressors. Research will need to integrate longitudinal investigations conducted in field settings with tightly controlled randomized clinical trials and complementary *in vitro* experiments. Investigations should move beyond solely examining changes in gut microbiota composition, and seek to define changes in the functional capacity and activity of the gut microbiota and other microorganisms (i.e., phage, virus, yeast and other fungi) by utilizing multi-omics approaches integrating genomic, transcriptomic and metabolomic data. Novel hypotheses generated from correlating these multi-omics data sets to environmental data and physiological, performance, and health outcomes will require targeted testing in the laboratory and the field. A key question for many of these studies should be to what extent any stress-induced changes in the gut microbiota persist and the functional consequences. Both human and animal studies suggest that perturbations such as antibiotic exposure and low-fiber intakes (Desai et al., 2016; Sonnenburg et al., 2016) may promote the loss of distinct bacterial populations (Jernberg et al., 2007; Jakobsson et al., 2010; Dethlefsen and Relman, 2011; Zaura et al., 2015), but whether the same is true for other stressors and the implications for human health remain uncertain. Findings from these investigations would be strengthened by developing technologies for non-invasively measuring the GI environment, and the distribution of microbes and their metabolites throughout the GI tract (e.g., Diaz Tartera et al., 2017; Kalantar-Zadeh et al., 2018). Such technologies would overcome limitations inherent with reliance on fecal samples which cannot capture differences in the composition and activity of gut microbes in different locations of the GI tract (e.g., proximal vs. distal colon, and mucosa vs. lumen). Current evidence of large interindividual variability in human gut microbiota composition (Falony et al., 2016) indicates that individual differences and the underpinning drivers of those differences will require consideration. However, this variability will also likely provide opportunities for developing algorithms for predicting responses to stress (e.g., Zeevi et al., 2015), and personalized strategies for favorably manipulating the gut microbiota (Zmora et al., 2016). Finally, as diet is a predominant, and potentially cost-effective mediator of gut microbiota composition and activity, future research should examine the extent to which poor nutrition contributes to deleterious stress responses within the gut microbiota, and aim to elucidate gut microbiota-targeted nutritional approaches that leverage the community’s tremendous functional potential to mitigate adverse stress responses.

## Author Contributions

All the authors contributed to the literature review, manuscript writing, and critical review of the manuscript. All the authors approved the final manuscript. JPK had primary responsibility for the final content.

## Disclaimer

The opinions or assertions contained herein are the private views of the author(s) and are not to be construed as official or as reflecting the views of the Army or the Department of Defense. Citation of commercial organizations or trade names in this report does not constitute an official Department of the Army endorsement or approval of the products or services of these organizations. Approved for public release (U18-137); distribution is unlimited.

## Conflict of Interest Statement

The authors declare that the research was conducted in the absence of any commercial or financial relationships that could be construed as a potential conflict of interest.

## Abbreviations

BCFA: branched-chain fatty acid
ETEC: entertoxigenic *Escherichia coli*
GI: gastrointestinal
HPA: hypothalamic-pituitary-adrenal
NDC: non-digestible carbohydrate
PAH: polycyclic aromatic hydrocarbons
PM: particulate matter
SCFA: short-chain fatty acid
SNS: sympathetic nervous system
TD: travelers’ diarrhea
